# The Role of Pericytes in Inner Ear Disorders: A Comprehensive Review

**DOI:** 10.3390/biology13100802

**Published:** 2024-10-08

**Authors:** Antonino Maniaci, Marilena Briglia, Fabio Allia, Giuseppe Montalbano, Giovanni Luca Romano, Mohamed Amine Zaouali, Dorra H’mida, Caterina Gagliano, Roberta Malaguarnera, Mario Lentini, Adriana Carol Eleonora Graziano, Giovanni Giurdanella

**Affiliations:** 1 Department of Medicine and Surgery, University of Enna “Kore”, 94100 Enna, Italy; antonino.maniaci@unikore.it (A.M.); marilena.briglia@unikore.it (M.B.); fabio.allia@unikore.it (F.A.); giovanniluca.romano@unikore.it (G.L.R.); caterina.gagliano@unikore.it (C.G.); roberta.malaguarnera@unikore.it (R.M.); giovanni.giurdanella@unikore.it (G.G.); 2 Department of Surgery, ENT Unit, Asp 7 Ragusa, 97100 Ragusa, Italy; 3 Zebrafish Neuromorphology Laboratory, Department of Veterinary Sciences, University of Messina, 98168 Messina, Italy; giuseppe.montalbano@unime.it; 4 Laboratory of Human Genome and Multifactorial Diseases (LR12ES07), Faculty of Pharmacy, University of Monastir, Avicenne Street, 5019 Monastir, Tunisia; mohamedamine.zaouali@isbm.u-monastir.tn; 5 Department of Cytogenetics and Reproductive Biology, Farhat Hached Hospital, 4021 Sousse, Tunisia; dorrahmidabenbrahim@gmail.com

**Keywords:** inner ear, pericytes, sensorineural hearing loss, pathophysiology, pericyte-target therapy

## Abstract

**Simple Summary:**

Hearing disorders, especially deafness, are highly debilitating diseases that negatively affect the quality of life. Despite the high incidence, the underlying pathophysiology of these disorders remains elusive, and current treatment options are often inadequate. Recent scientific evidence demonstrates the importance of the key role of pericytes as vascular mural cells specialized in maintaining the integrity and functioning of microvasculature. Understanding their activity in inner ear disorders can provide useful knowledge about the pathophysiology of these conditions and ensure the development of new diagnostic strategies. The aim of this comprehensive review is to provide a detailed description of the involvement of these cells in disorders affecting the inner ear by analyzing the mechanisms that cause them. In addition, based on current knowledge, focus is also placed on future prospects and new targeted therapeutic strategies.

**Abstract:**

Inner ear disorders, including sensorineural hearing loss, Meniere’s disease, and vestibular neuritis, are prevalent conditions that significantly impact the quality of life. Despite their high incidence, the underlying pathophysiology of these disorders remains elusive, and current treatment options are often inadequate. Emerging evidence suggests that pericytes, a type of vascular mural cell specialized to maintain the integrity and function of the microvasculature, may play a crucial role in the development and progression of inner ear disorders. The pericytes are present in the microvasculature of both the cochlea and the vestibular system, where they regulate blood flow, maintain the blood–labyrinth barrier, facilitate angiogenesis, and provide trophic support to neurons. Understanding their role in inner ear disorders may provide valuable insights into the pathophysiology of these conditions and lead to the development of novel diagnostic and therapeutic strategies, improving the standard of living. This comprehensive review aims to provide a detailed overview of the role of pericytes in inner ear disorders, highlighting the anatomy and physiology in the microvasculature, and analyzing the mechanisms that contribute to the development of the disorders. Furthermore, we explore the potential pericyte-targeted therapies, including antioxidant, anti-inflammatory, and angiogenic approaches, as well as gene therapy strategies.

## 1. Introduction

Inner ear disorders are a group of conditions that affect the delicate structures responsible for hearing and balance [1,2]. These disorders can lead to various symptoms, including hearing loss, tinnitus, vertigo, and dizziness. 

Some common inner ear disorders include sensorineural hearing loss, Meniere’s disease, vestibular neuritis, and benign paroxysmal positional vertigo (BPPV) [3]. These conditions can significantly impact an individual’s quality of life, leading to communication difficulties, social isolation, and an increased risk of falls and accidents [4]. 

**Figure 1:**
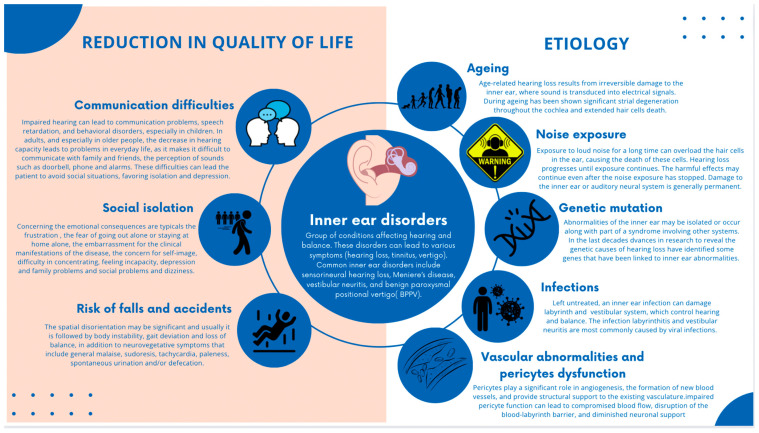
Graphical representation of inner ear disorders and related conditions that significantly affect the quality of life, including the multifactorial etiology of inner ear disorders.

The etiology of inner ear disorders is multifactorial, with factors such as aging, noise exposure, genetic mutations, infections, and vascular abnormalities contributing to their development [5,6,7,8] (Figure 1). 

Recently, a growing interest in the role of pericytes, in the pathophysiology of inner ear disorders emerged [9]. Pericytes are specialized cells that wrap around the endothelial cells of capillaries and play a crucial role in maintaining the integrity and function of the microvasculature [10,11]. 

In the inner ear, pericytes are found in the microvasculature of both the cochlea and the vestibular system. The functions of pericytes are crucial and encompass various vital roles within the features of the vessels justifying their high density within some vascular areas [11,12,13]. Firstly, pericytes possess contractile properties that allow them to regulate blood flow by modulating capillary diameter, thereby responding to local metabolic demands [14]. Secondly, pericytes actively participate in maintaining the integrity of the blood–labyrinth barrier, a crucial structure that safeguards the inner ear’s unique ionic composition [15,16]. Their contribution to the formation and preservation of this barrier is essential in preserving optimal auditory and vestibular function [17]. 

Furthermore, pericytes play a significant role in angiogenesis, the formation of new blood vessels, and provide structural support to the existing vasculature [18,19,20]. This dual function aids in ensuring adequate blood supply to tissues and organ systems, promoting their proper functioning. 

Moreover, studies have indicated that pericytes offer neuronal support by providing trophic factors to neurons [21]. This support is essential for maintaining the health and function of neurons within the intricate network of the inner ear [22]. 

However, pericyte dysfunction can have profound implications for various pathological conditions such as neurodegenerative diseases, diabetic retinopathy, and tumor angiogenesis [23,24,25]. In the context of inner ear disorders, impaired pericyte function can lead to compromised blood flow, disruption of the blood–labyrinth barrier, and diminished neuronal support [22]. These consequences contribute significantly to the development and progression of inner ear pathological conditions as observed in inflammation, Meniere’s disease, genetic hearing loss, and age or loud-sound related impairment of mouse cochlea, emphasizing the importance of pericyte function in maintaining inner ear health [15,22,26,27,28]. This comprehensive review aims to provide an in-depth overview of the role of pericytes in inner ear disorders focusing on the distribution and function of pericytes in the cochlear and vestibular microvasculature, and the mechanisms by which pericyte dysfunction may contribute to various inner ear disorders. Furthermore, we explore potential pericyte-targeted therapies and discuss current research and future directions in this field.

## 2. Distribution and Function of Pericytes in the Cochlear and Vestibular Microvasculature

Pericytes are a type of mural cell that are closely associated with the endothelial cells of capillaries and play a crucial role in regulating blood flow, vascular permeability, and angiogenesis [29,30,31]. 

In the cochlea, pericytes were observed in a high density within the microvasculature of the spiral ganglion region, with a pericyte-to-endothelial cell ratio of approximately 1:1 [32]. This high ratio suggests that pericytes play a particularly important role in regulating blood flow and maintaining the integrity of the blood–labyrinth barrier in the cochlea [33].

Recent studies based on advanced imaging techniques, such as two-photon microscopy and electron microscopy, have provided new insights into the morphology and distribution of pericytes in the cochlear microvasculature of the stria vascularis. This latter exerts a pivotal role in maintaining the blood–labyrinth barrier integrity and cochlear functions. These studies have shown that pericytes are not uniformly distributed along the length of capillaries but are concentrated at branch points and in regions of high curvature [34,35]. This strategic positioning allows pericytes to regulate blood flow and respond to local changes in oxygen and nutrient demand [36].

In addition to their structural and regulatory functions, pericytes in the cochlea have been shown to actively communicate with spiral ganglion neurons (SGNs) through the release of extracellular vesicles (EVs) [21]. These EVs, which include exosomes and microvesicles, contain a variety of signaling molecules, growth factors, and genetic material that can influence the behavior and survival of SGNs [37]. 

One of the key molecules found in pericyte-derived EVs is vascular endothelial growth factor-A (VEGF-A), a potent angiogenic factor that has been shown to promote SGN survival and neurite outgrowth in vitro and in vivo [38]. 

Other factors found in pericyte-derived EVs include brain-derived neurotrophic factor (BDNF), neurotrophin-3 (NT-3), and platelet-derived growth factor (PDGF), all of which have been implicated in the maintenance and repair of the auditory nerve [39,40]. 

The presence of pericyte-derived EVs within the cell bodies of SGNs suggests a direct communication between these two cell types and highlights the importance of pericytes in maintaining the health and function of the auditory nerve [41]. This communication is thought to be bidirectional, with SGNs also releasing factors that can influence pericyte behavior and survival. For example, SGNs were shown to express PDGF receptor β (PDGFRβ), which can bind to PDGF released by pericytes and promote their recruitment and proliferation [42]. 

In the vestibular system, pericytes are also found in close association with the microvasculature of the semicircular canals and otolith organs [43]. While less is known about the specific distribution and function of pericytes in the vestibular system compared to the cochlea, recent studies suggest that they play a similar role in regulating blood flow and providing support to the vestibular hair cells and nerve fibers [44]. 

For example, a study using a mouse model of vestibular dysfunction showed that pericyte detachment and loss were associated with reduced capillary density and increased permeability of the blood–labyrinth barrier in the vestibular end organs [45]. This suggests that pericytes are important for maintaining the integrity of the vestibular microvasculature and preventing the entry of potentially harmful substances into the inner ear fluids. 

**Figure 2:**
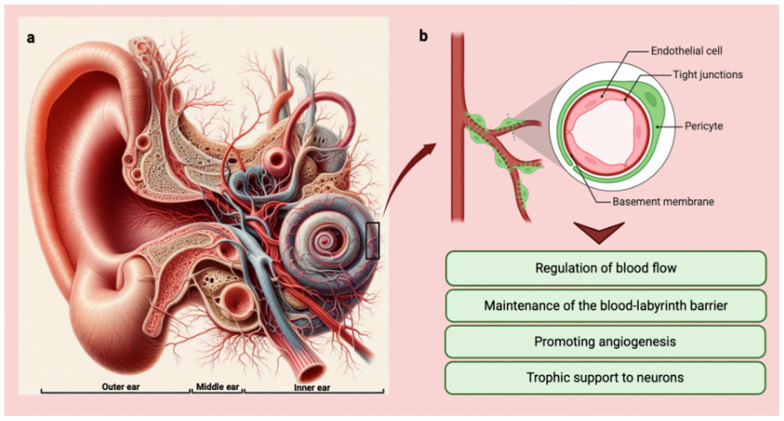
(**a**) Anatomy of the ear with vessel distribution and organization (Copilot image creator, Microsoft designer, Bing, www.bing.com, accessed on 1 September 2024). (**b**) Graphic showing pericytes and their main functions in the inner ear (BioRender: Scientific Image and Illustration Software, Toronto, Canada, www.biorender.com, accessed on 1 September 2024).

Moreover, pericytes of the vestibular system may also communicate with vestibular hair cells and afferent nerve fibers through the release of EVs and other signaling molecules [46]. While more research is needed to elucidate the specific functions of pericytes in the vestibular system, it is likely that they play a similar role to that seen in the cochlea, providing trophic support and promoting the survival and function of the cells responsible for balance and spatial orientation [16] (Figure 2). 

## 3. Pericyte–Endothelial Cell Interactions and Response to Injury and Stress

Pericytes and endothelial cells have a close spatial and functional relationship within the microvasculature of the inner ear [47]. Pericytes are embedded within the basement membrane of capillaries in a direct contact with endothelial cells through specialized junctions known as peg-and-socket contacts [48]. These contacts allow for bidirectional communication between the two cell types, enabling pericytes to regulate endothelial cell function and vice versa. 

Pericytes have been shown to regulate endothelial cell proliferation, migration, and differentiation, as well as to modulate vascular permeability and blood flow in response to local cues [49]. In turn, endothelial cells can influence pericyte recruitment, proliferation, and differentiation through the release of growth factors such as platelet-derived growth factor-B (PDGF-B) and transforming growth factor-β (TGF-β) [50]. 

A disruption of this delicate balance between pericytes and endothelial cells can lead to vascular dysfunction and subsequent hearing and balance disorders. Pericytes have been shown to be particularly sensitive to injury and stress, and their dysfunction and consequent loss have been implicated in a variety of neurodegenerative [51] and vascular disorders [52,53], including age-related hearing loss [54]. 

In the cochlea, pericyte loss has been observed in animal models of noise-induced hearing loss and age-related hearing loss, suggesting that these cells may be an early target of damage in the aging or noise-exposed ear [55,56]. Using an inducible pericyte depletion mouse model, researchers have demonstrated that the targeted ablation of pericytes leads to a significant reduction in vascular density and volume within the spiral ganglion region, as well as a decrease in the number of SGNs and impaired hearing sensitivity [22]. These findings underline the critical role of pericytes in maintaining the health and function of the cochlear microvasculature and the auditory nerve [57]. 

Pericytes have also been shown to respond to injury and stress by undergoing phenotypic changes and participating in the inflammatory response [58,59]. In response to tissue damage or hypoxia, pericytes can detach from the capillary wall and migrate into the surrounding tissue, where they can differentiate into myofibroblasts and contribute to fibrosis and scar formation [60,61]. Additionally, pericytes have been shown to express a variety of inflammatory mediators, such as interleukin-6 (IL-6) and monocyte chemoattractant protein-1 (MCP-1), which can attract immune cells to the site of injury and promote further damage [62,63]. 

## 4. Inner Ear Disorders Associated with Pericyte Dysfunction

### 4.1. Hearing Disorders

Pericyte dysfunction was observed in a variety of inner ear disorders, ranging from sensorineural hearing loss to vestibular disorders and tinnitus [17,44]. While the specific mechanisms by which pericyte dysfunction contributes to these disorders are still being elucidated [29], it is clear that these cells play a critical role in maintaining the health and function of the inner ear microvasculature and the cells that depend on it [64]. Pericyte dysfunction has been implicated in several types of sensorineural hearing loss (SNHL), including age-related hearing loss, noise-induced hearing loss, and genetic hearing loss [27,28]. Age-related hearing loss (ARHL), also known as presbycusis, is a gradual loss of hearing that occurs with aging, and it is one of the most common types of hearing loss that affects millions of people worldwide [65]. 

Recent studies have shown that pericyte loss and dysfunction may contribute to the pathogenesis of ARHL [66]. In animal models of aging, pericyte loss was observed in the cochlear microvasculature, particularly in the stria vascularis and spiral ligament [67,68]. This loss is associated with reduced capillary density, increased vascular permeability, and decreased expression of tight junction proteins, all of which can lead to a disruption of the blood–labyrinth barrier and impaired ion homeostasis in the inner ear fluids [69,70]. 

Pericyte loss was also associated with decreased expression of neurotrophic factors, such as BDNF and NT-3, which represent important mediators for the survival and function of spiral ganglion neurons [39,40]. These changes may contribute to the progressive loss of hair cells and auditory nerve fibers, which is a characteristic sign of ARHL. 

In addition, pericyte dysfunction has been implicated in the pathogenesis of noise-induced hearing loss (NIHL), particularly in the early stages of the disorder [27]. In animal models of NIHL, pericyte loss and detachment from the capillary wall were observed in the cochlear microvasculature within hours of noise exposure [71]. These are followed by increased vascular permeability, leukocyte infiltration, and oxidative stress, contributing to hair cell damage and auditory nerve degeneration [72]. Pericyte-derived EVs have also been shown to be altered in response to noise exposure, with changes in the levels of pro-inflammatory cytokines and chemokines that may contribute to the inflammatory response in the inner ear [21,73]. 

Genetic hearing loss is a type of hearing loss that results from mutations in genes that are important for the development and function of the inner ear [74,75]. Pericyte dysfunction is related to some forms of genetic hearing loss, particularly those associated with vascular abnormalities in the cochlea [76]. Mutations in the gene encoding norrin, a protein that is important for vascular development and pericyte recruitment, were associated with a type of progressive hearing loss known as Norrie disease [77,78]. 

In animal models of Norrie disease, pericyte loss and vascular abnormalities have been observed in the cochlear microvasculature, leading to impaired blood flow and oxidative stress in the inner ear [26,79]. Similarly, mutations in the gene encoding PDGF receptor β (PDGFRβ), which is expressed by pericytes and is important for their recruitment and survival, have been associated with a type of autosomal dominant hearing loss known as DFNA66 [80,81]. These findings suggest that pericyte dysfunction may be a common mechanism underlying some forms of genetic hearing loss. 

### 4.2. Vestibular Disorders

Pericyte abnormalities have been implicated in several types of vestibular disorders, including Meniere’s disease, vestibular neuritis, and benign paroxysmal positional vertigo (BPPV) [3,9,16,82]. Meniere’s disease is a chronic condition characterized by episodes of vertigo, fluctuating hearing loss, tinnitus, and aural fullness [83]. While the exact cause of Meniere’s disease is unknown, it has been suspected of involving an alteration in the fluid balance of the inner ear, leading to endolymphatic hydrops [84,85,86]. 

Recent studies have suggested that pericyte dysfunction may contribute to the pathogenesis of Meniere’s disease by altering the permeability of the blood–labyrinth barrier and allowing for the entry of inflammatory mediators and other potentially harmful substances into the inner ear fluids [87]. 

In animal models of endolymphatic hydrops [88], pericyte loss and increased vascular permeability have been observed in the cochlear and vestibular microvasculature, along with changes in the expression of tight junction proteins and inflammatory cytokines [87]. An ultrastructural investigation of the pericytes in Meniere’s disease displayed an altered organization. They showed a thinning of cellular processes that do not evenly coat the wall of the vessels [17]. The disruption of the perivascular basal membrane surrounding the endothelium causes severe edematous abnormalities, as well as excessive stromal edema with large vacuoles [87]. Cochlear hydrops typifies Meniere’s disease [86,89]. These pieces of evidence show that pericytes play a key role in regulating blood barrier integrity and controlling vascular permeability while pericyte pathology could be a contributing factor to BLB rupture causing cochlear edema. 

These findings suggest that targeting pericyte function may be a potential therapeutic strategy for Meniere’s disease. 

Vestibular neuritis is a condition characterized by sudden onset of severe vertigo, nausea, and imbalance, often accompanied by hearing loss and tinnitus and is caused by inflammation of the vestibular nerve, possibly due to viral infection or autoimmune disease [90]. Pericyte dysfunction was correlated with the pathogenesis of vestibular neuritis, particularly in the context of viral infection [91]. 

In animal models of vestibular neuritis, pericyte loss and increased vascular permeability were observed in the vestibular end organs and nerve, along with infiltration of immune cells and increased expression of pro-inflammatory cytokines [43,92,93]. In order to better understand the pathogenesis of vestibular neuronitis, Hirata et al. conducted a sero-virological study on patients diagnosed with vestibular neuronitis according to diagnostic criteria. A significant change in the serum viral titre of antibodies (HSV, CMV, EBV, rubella, adeno) was found in many cases. 

Data indicate that the infections caused by these viruses may have a correlation with the onset of dizziness and vestibular neuritis [94]. Particularly in the otolaryngology field, herpesvirus infection mainly causes hearing loss and vestibular neuritis. It is considered the primary assumption regarding the pathogenesis of vestibular neuritis [95]. 

Regarding the recent coronavirus pandemic, several clinical studies, case reports, original research, and systematic reviews described the impact of SARS-CoV-2 on otoneurological dysfunction. SARS-CoV-2 was shown to cause severe damage to the vestibular system. Moreover, to better understand and verify the short- and long-term effects of COVID-19 on vestibular function, additional translational and clinical studies will be required [95,96,97,98,99,100]. 

Pericyte-derived EVs were also shown to be altered in response to viral infection, with changes in the levels of antiviral and pro-inflammatory mediators that may contribute to the immune response in the inner ear. BPPV is a common vestibular disorder characterized by brief episodes of vertigo triggered by changes in the head position [101]. 

While the role of pericyte dysfunction in BPPV is less clear than in other vestibular disorders, some studies have suggested that changes in the microvasculature of the vestibular end organs may contribute to the development of BPPV [102]. In animal models of aging, pericyte loss and decreased capillary density were observed in the vestibular end organs, along with changes in the composition and mechanical properties of the otoconia [103]. 

These changes may increase the susceptibility of the otoconia to displacement and contribute to the development of BPPV in older individuals [104]. Several studies have correlated the tinnitus incidence with the perception of sound in the absence of an external auditory stimulus [105,106,107]. Pericyte dysfunction was noticed in the pathogenesis of tinnitus, particularly in the context of cochlear injury and inflammation [108]. 

### 4.3. Sensory Neurologic Disorders

In animal models of tinnitus, pericyte loss and increased vascular permeability have been observed in the cochlear microvasculature, along with changes in the expression of neurotrophic factors and inflammatory mediators [109,110,111]. Pericyte-derived EVs were also shown to be altered in response to cochlear injury, with changes in the levels of pro-inflammatory cytokines and growth factors, contributing to the development of tinnitus [21]. These findings suggest that targeting pericyte function may be a potential therapeutic strategy for tinnitus, particularly in cases associated with cochlear injury or inflammation [112]. 

The Auditory Neuropathy Spectrum Disorder (ANSD) is a type of hearing disorder characterized by impaired auditory nerve function despite normal hair cell function. It can be caused by a variety of factors, including genetic mutations, prematurity, and exposure to certain toxins and medications [113,114]. In a mouse model of ANSD caused by mutation in the gene encoding pejvakin (a protein important for auditory nerve function), pericyte loss and decreased capillary density were observed in the cochlear microvasculature, along with impaired auditory nerve function and degeneration [115]. 

Similarly, in a mouse model of ANSD caused by ouabain exposure (a toxin that selectively damages auditory nerve fibers), pericyte dysfunction and vascular abnormalities were observed in the cochlear microvasculature, along with selective degeneration of auditory nerve fibers [116]. 

The mechanisms by which pericyte dysfunction contributes to ANSD are still being elucidated but may involve several pathways [117]. 

First, pericyte loss and vascular abnormalities may lead to decreased blood flow and oxygen delivery to the auditory nerve, leading to oxidative stress and nerve fiber degeneration [118,119]. Second, pericyte dysfunction may alter the permeability of the blood–labyrinth barrier, allowing for the entry of toxins or inflammatory mediators that can damage the auditory nerve [44,120]. Third, pericyte dysfunction may impair the production and release of neurotrophic factors, such as BDNF and NT-3, which are important for the survival and function of auditory nerve fibers [121,122]. 

Finally, pericyte-derived EVs may play a role in the pathogenesis of ANSD by altering the levels of pro-inflammatory cytokines, growth factors, and other signaling molecules that can influence auditory nerve function and survival [21,41]. Despite these potential mechanisms, the role of pericyte dysfunction in ANSD remains an active area of research, and more studies are needed to fully understand the complex interactions between pericytes, endothelial cells, and auditory nerve fibers in this disorder [113,117,123]. 

However, the identification of pericyte dysfunction as a potential contributor to ANSD suggests that targeting pericyte function may be a promising therapeutic strategy for this disorder, particularly in cases associated with vascular abnormalities or impaired neurotrophic support [61,120]. 

## 5. Inflammation, Oxidative Stress, and Genetic Signaling of Pericyte Dysfunction

Inflammation is a complex biological response to tissue injury or infection that involves the recruitment and activation of immune cells, the production of cytokines and chemokines, and the remodeling of the extracellular matrix [124,125]. 

**Figure 3:**
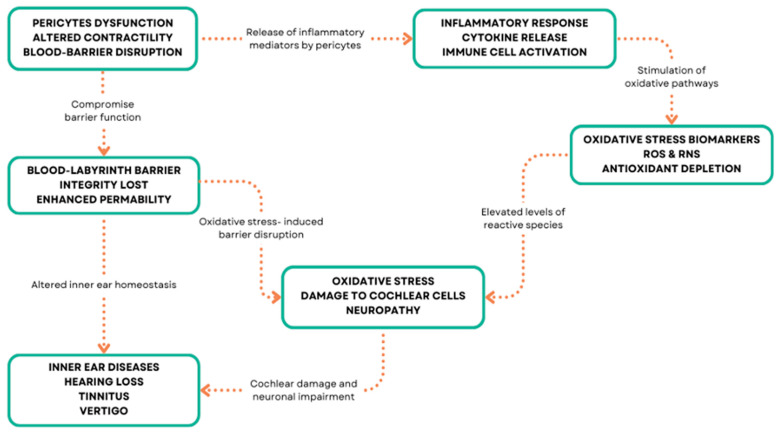
Pericyte molecular patterns and inner ear diseases.

In the inner ear, inflammation has been implicated in various disorders, including autoimmune inner ear disease, labyrinthitis, and vestibular neuritis [126,127,128] (Figure 3). Inflammatory-mediated pericytes dysfunction can arise from various mechanisms, including oxidative stress, ischemia, hypoxia, and genetic mutations in inner ear disorders [129,130]. These mechanisms can lead to pericyte loss, detachment from the capillary wall, and impaired function, resulting in vascular abnormalities and compromised inner ear homeostasis [131]. Oxidative stress occurs when there is an imbalance between the production of reactive oxygen species (ROS) and the ability of cells to detoxify them or repair the resulting damage [132,133] (Figure 3). 

In the inner ear, oxidative stress has been implicated in various disorders, including age-related hearing loss, noise-induced hearing loss, and ototoxicity [134,135,136]. Pericytes are particularly susceptible to oxidative stress due to their high metabolic activity and close proximity to endothelial cells, which are a major source of ROS [137,138]. Under conditions of oxidative stress, pericytes can undergo morphological and functional changes, such as cell contraction, migration, and detachment from the capillary wall [12,139]. These changes can lead to increased vascular permeability, reduced blood flow, and impaired nutrient and oxygen delivery to the inner ear tissues [140].

Additionally, oxidative stress can induce pericyte apoptosis and senescence, leading to a reduction in pericyte coverage and density [141]. Pericytes can also contribute to oxidative stress by producing ROS themselves, particularly under conditions of hypoxia or ischemia [142]. In vitro studies have shown that pericytes can generate superoxide anion and hydrogen peroxide, which can further exacerbate oxidative damage to nearby cells [143]. Pericytes are important regulators of inflammation in the inner ear, as they can both respond to and produce inflammatory mediators [144]. In response to inflammatory stimuli, such as lipopolysaccharide or tumor necrosis factor-α (TNF-α), pericytes can upregulate the expression of adhesion molecules, such as intercellular adhesion molecule-1 (ICAM-1) and vascular cell adhesion molecule-1 (VCAM-1), which facilitate the recruitment and extravasation of leukocytes [145,146]. Pericytes can also produce pro-inflammatory cytokines, such as interleukin-1β (IL-1β), interleukin-6 (IL-6), and TNF-α, which can amplify the inflammatory response and contribute to tissue damage [147,148]. 

Conversely, pericytes can also have anti-inflammatory effects in the inner ear, particularly through the production of transforming growth factor-β (TGF-β) [149]. 

TGF-β is a potent immunomodulatory cytokine that can inhibit the proliferation and activation of lymphocytes, reduce the production of pro-inflammatory cytokines, and promote the resolution of inflammation [150,151,152]. Pericyte-derived TGF-β has been shown to protect against noise-induced hearing loss and age-related hearing loss in animal models, possibly by attenuating the inflammatory response and promoting the survival of hair cells and spiral ganglion neurons [153]. Ischemia and hypoxia refer to conditions of reduced blood flow and oxygen delivery to tissues, respectively. 

Pericytes are highly sensitive to changes in oxygen tension and can respond to hypoxia by regulating capillary diameter and blood flow [29]. Under hypoxic conditions, pericytes can contract and narrow the capillary lumen, reducing blood flow and exacerbating tissue ischemia [154,155]. This response is mediated by the activation of hypoxia-inducible factor-1α (HIF-1α), a transcription factor that regulates the expression of genes involved in angiogenesis, metabolism, and cell survival [71,156]. Prolonged or severe hypoxia can lead to pericyte dysfunction and loss, as well as endothelial cell damage and capillary regression [157]. 

**Figure 4:**
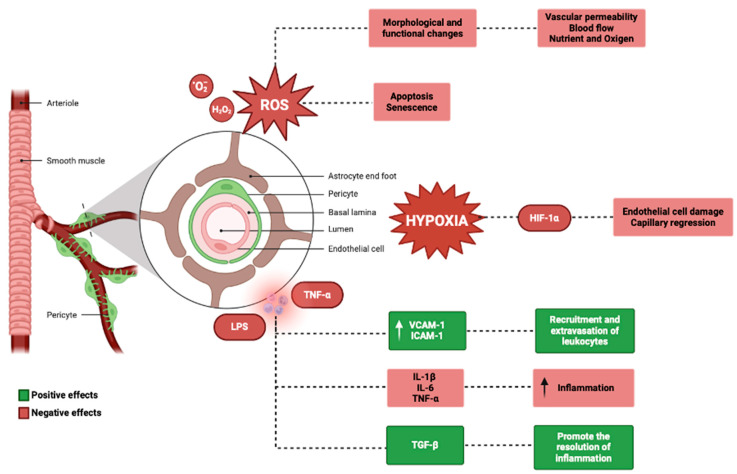
Genetics and physiological mechanisms in the pericytes (BioRender: Scientific Image and Illustration Software, www.biorender.com, accessed on 1 September 2024).

In the inner ear, ischemia-induced pericyte dysfunction has been shown to contribute to the pathogenesis of sudden sensorineural hearing loss, possibly by impairing the integrity of the blood–labyrinth barrier and allowing for the entry of inflammatory mediators and other harmful substances into inner ear fluids [158] (Figure 4).

Genetic mutations may impact pericyte function in the inner ear by altering the expression or function of genes involved in pericyte development, recruitment, or signaling [159,160]. These mutations can be inherited in a Mendelian fashion or can arise de novo, and can lead to a variety of inner ear disorders, including hereditary hearing loss and vestibular dysfunction [161,162]. A notable genetic mutation affecting pericyte function targets the PDGFRB gene, which encodes the platelet-derived growth factor receptor-β (PDGFR-β). PDGFR-β is a tyrosine kinase receptor expressed by pericytes that exerts an essential role for their recruitment and survival [163,164,165]. Mutations in PDGFRB have been associated with autosomal dominant familial brain calcification, a rare neurological disorder that can also present with hearing loss and vestibular dysfunction [166,167]. In animal models, the deletion of PDGFRB in pericytes leads to pericyte loss, capillary regression, and impaired blood–brain barrier function, suggesting that PDGFR-β signaling is critical for pericyte maintenance and vascular stability in the inner ear [168,169]. Another example of a genetic mutation affecting pericyte function is the mutation in the COCH gene, which encodes cochlin, a protein that is highly expressed in the inner ear and is involved in the regulation of extracellular matrix homeostasis [170,171,172,173]. Mutations in COCH were associated with autosomal dominant nonsyndromic hearing loss and vestibular dysfunction, possibly by altering the interaction between cochlin and type II collagen in the inner ear [174,175,176,177]. 

Recent studies have shown that cochlin is also expressed by pericytes of the inner ear and may regulate their adhesion and migration, suggesting that COCH mutations may impact pericyte function and contribute to the pathogenesis of inner ear disorders [178]. The identification of genetic mutations affecting pericyte function in the inner ear may provide new insights into the molecular mechanisms underlying inherited inner ear disorders and may guide the development of targeted therapies [179,180].

## 6. Pericyte-Targeted Therapies for Inner Ear Disorders

Given the critical role of pericytes in maintaining the health and function of the microvasculature, targeting pericyte dysfunction has emerged as a promising therapeutic strategy for inner ear disorders [137]. Pericyte-targeted therapies aim to prevent pericyte detachment and loss or their impaired function, thereby preserving vascular stability and promoting inner ear homeostasis [33]. These therapies can be broadly divided into antioxidant therapies, anti-inflammatory therapies, angiogenic therapies, and gene therapy approaches [181]. Antioxidant therapies aim to reduce oxidative stress in the inner ear by scavenging ROS or enhancing the endogenous antioxidant defense system [143,182]. As pericytes are particularly susceptible to oxidative damage, antioxidant therapies may help to protect pericytes and preserve their function in the context of inner ear disorders [183]. One promising antioxidant therapy for inner ear disorders is the use of N-acetylcysteine (NAC), a glutathione precursor that has been shown to attenuate pericyte loss and improve capillary density in animal models of noise-induced hearing loss and age-related hearing loss [184,185,186,187]. NAC can scavenge ROS directly or can enhance the production of glutathione, a major endogenous antioxidant that protects inner ear hair cells against oxidative damage [188]. In addition to its antioxidant effects, NAC has been shown to have anti-inflammatory and anti-apoptotic properties, which may further contribute to its protective effects on pericytes and other inner ear cells [189]. Other antioxidant therapies that have been explored for inner ear disorders include the use of vitamins C and E, coenzyme Q10, and polyphenolic compounds such as resveratrol and curcumin [130,190,191,192,193]. A study by Yang et al. found that the administration of low doses of resveratrol to mice with age-related hearing loss led to a significant increase in the number of pericytes and a significant reduction in oxidative stress and inflammation [194]. These compounds can scavenge ROS, chelate metal ions, or modulate the expression of antioxidant enzymes, thereby reducing oxidative stress and preserving pericyte function [195]. Anti-inflammatory therapies have been shown to be promising in the inner ear. 

As pericytes can produce inflammatory mediators, anti-inflammatory therapies may help to attenuate pericytes’ pathological damage and preserve their function in inner ear inflammation [196]. Corticosteroids can reduce the production of pro-inflammatory cytokines, such as TNF-α and IL-1β, and can inhibit the recruitment and activation of immune cells [197,198]. In animal models of noise-induced hearing loss and autoimmune inner ear disease, the local or systemic administration of corticosteroids was able to attenuate pericyte loss, reduce vascular permeability, and improve hearing function [199,200]. 

However, the long-term use of corticosteroids can be associated with adverse effects, such as immunosuppression and metabolic disturbances, which may limit their clinical application [201]. 

Another potential anti-inflammatory therapy for inner ear disorders is the use of TNF-α inhibitors, such as etanercept or infliximab [202,203]. TNF-α is a potent pro-inflammatory cytokine that can activate pericytes and induce their production of other inflammatory mediators [23,204]. TNF-α inhibitors can block the binding of TNF-α to its receptors, thereby attenuating its pro-inflammatory effects [205]. 

In animal models of noise-induced hearing loss and cisplatin-induced ototoxicity, the local or systemic administration of TNF-α inhibitors attenuated pericyte loss, reduced oxidative stress, and improved hearing function [206,207]. However, the use of TNF-α inhibitors in human inner ear disorders is still limited, and more clinical trials are needed to evaluate their safety and efficacy. 

In addition to these pharmacological approaches, the modulation of pericyte-derived anti-inflammatory mediators, such as TGF-β, may also be a promising strategy for inner ear disorders [149]. TGF-β is a potent immunomodulatory cytokine that can inhibit the proliferation and activation of lymphocytes, reduce the production of pro-inflammatory cytokines, and promote the resolution of inflammation [152]. The delivery of exogenous TGF-β or the enhancement of endogenous TGF-β signaling in pericytes may help to attenuate the inflammatory response and preserve pericyte function in the inner ear [208]. However, the therapeutic potential of TGF-β modulation in human inner ear disorders remains to be explored. Angiogenic therapies aim to promote the growth and regeneration of blood vessels in the inner ear by delivering pro-angiogenic factors or modulating the expression of angiogenic regulators [209]. As pericytes play a critical role in angiogenesis and vascular stabilization, angiogenic therapies may help to recruit pericytes and preserve their function in the context of inner ear vascular damage or insufficiency [210]. 

One promising angiogenic therapy for inner ear disorders is the delivery of vascular endothelial growth factor (VEGF), a potent pro-angiogenic factor that can stimulate endothelial cell proliferation, migration, and tube formation [211]. VEGF can also recruit pericytes and promote their adhesion and survival, thereby stabilizing the newly formed vessels. In animal models of noise-induced hearing loss and vestibular schwannoma, the local or systemic administration of VEGF increased capillary density, attenuated pericyte loss, and improved hearing or vestibular function [38,212]. However, the therapeutic use of VEGF in human inner ear disorders is still limited by its potential side effects, such as vascular leakage and edema. Moreover, recent studies support the hypothesis that VEGF mediates increased vascular permeability in an in vitro model of cisplatin-induced ototoxicity [213]. Another potential angiogenic therapy for inner ear disorders is the modulation of hypoxia-inducible factor-1α (HIF-1α), a transcription factor that regulates the expression of pro-angiogenic genes in response to hypoxia [214,215]. HIF-1α can induce the expression of VEGF, erythropoietin, and other factors that promote angiogenesis and cell survival. The pharmacological activation of HIF-1α or the inhibition of its degradation may enhance the angiogenesis and preserve pericyte function in the inner ear [215,216]. In addition to these molecular approaches, cell-based therapies using pericyte progenitor cells or mesenchymal stem cells (MSCs) may also be a promising strategy for inner ear disorders [217,218,219,220]. Pericyte progenitor cells and MSCs can differentiate into pericytes and secrete pro-angiogenic and anti-inflammatory factors, thereby promoting vascular regeneration and attenuating tissue damage [219,221]. In animal models of noise-induced hearing loss and age-related hearing loss, the transplantation of MSCs or pericyte progenitor cells has been shown to increase capillary density, attenuate pericyte loss, and improve hearing function [222,223]. 

However, the clinical translation of cell-based therapies for inner ear disorders is still limited by the challenges in cell delivery, survival, and differentiation. Gene therapy approaches aim to correct the genetic defects [224] or enhance the expression of protective genes in pericytes or other inner ear cells [225,226]. As genetic mutations can impact pericyte function and contribute to the pathogenesis of inner ear disorders, gene therapy may provide a targeted and long-lasting solution for these conditions [225]. The use of adeno-associated virus (AAV) vectors is considered a promising gene therapy approach to inner ear disorders in order to deliver therapeutic genes to pericytes or other inner ear cells. AAV vectors are non-pathogenic, can transduce both dividing and non-dividing cells, and can achieve long-term gene expression [227,228]. 

**Figure 5:**
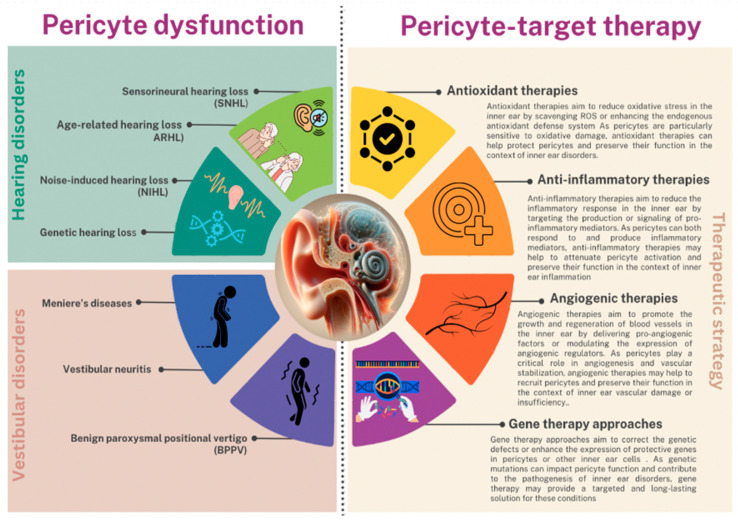
Representation of pericyte dysfunction consequences and promising therapeutic strategies for inner ear disorders.

In animal models of hereditary hearing loss and vestibular dysfunction, the local or systemic administration of AAV vectors carrying the wild-type gene or a compensatory gene has been shown to attenuate pericyte loss, improve vascular function, and restore hearing or balance [229,230]. However, the clinical translation of AAV-mediated gene therapy for inner ear disorders is still limited by the challenges in vector design, delivery, and safety [231]. Another potential gene therapy approach to inner ear disorders is based on the use of CRISPR-Cas9 technology to correct the genetic mutations in pericytes or other inner ear cells [232]. CRISPR-Cas9 is a powerful gene-editing tool that can introduce precise modifications in the genome, such as the correction of disease-causing mutations or the insertion of protective genes [233,234]. In animal models of hereditary hearing loss, the local delivery of CRISPR-Cas9 components targeting the mutated gene has been shown to restore hearing function and preserve inner ear morphology [235,236]. However, the therapeutic application of CRISPR-Cas9 in human inner ear disorders is still limited by the challenges in delivery, specificity, and potential off-target effects. In addition to these gene therapy approaches, the modulation of microRNAs (miRNAs) may also be a promising strategy for inner ear disorders [237]. miRNAs are small non-coding RNAs that can regulate gene expression post-transcriptionally by binding to complementary sequences in the target mRNA. Pericytes express a variety of miRNAs that can regulate their function and survival, such as miR-132 and miR-145 [238]. The delivery of miRNA mimics or inhibitors to pericytes may help to enhance their protective effects or attenuate their pathogenic responses in the context of inner ear disorders [239,240]. However, the therapeutic potential of miRNA modulation in human inner ear disorders remains to be better explored (Figure 5).

## 7. Animal Models for Studying Pericytes in Inner Ear Disorders

Animal models are essential tools for studying the role of pericytes in inner ear disorders and for testing the efficacy and safety of pericyte-targeted therapies [241]. These models can recapitulate the key features of human inner ear disorders, such as hearing loss, vestibular dysfunction, and vascular abnormalities, and can provide valuable insights into the underlying mechanisms of pericyte dysfunction [241,242]. The most commonly used animal models for studying pericytes in inner ear disorders are mouse models, zebrafish models, and in vitro models [243,244,245].

### 7.1. Mouse Models

Mouse models are the most widely used animal models for studying pericytes in inner ear disorders due to their genetic similarity to humans, their well-characterized genome, and the availability of various genetic tools for manipulating pericyte function [246]. Several mouse models have been developed to study the role of pericytes in different types of inner ear disorders, such as noise-induced hearing loss, age-related hearing loss, and hereditary hearing loss [44,55,247].

One example of a mouse model for studying pericytes in noise-induced hearing loss is the Pdgfrb-CreERT2;Rosa26-tdTomato mouse line, which allows for the specific labeling and tracking of pericytes in the inner ear [22]. Using this model, researchers have shown that exposure to loud noise can cause pericyte loss, vascular dysfunction, and hearing impairment, and that these effects can be attenuated by antioxidant or anti-inflammatory therapies [22,248]. 

Another interesting mouse model for studying pericytes in age-related hearing loss is the Cdh23ahl mouse line, which carries a mutation in the cadherin 23 gene and develops progressive hearing loss similar to that seen in older humans [249,250]. Using this model, researchers have shown that pericyte loss and vascular degeneration occur early in the aging process and precede the onset of hearing loss, suggesting that pericyte dysfunction may be a driving factor in age-related hearing loss [250,251]. In addition to these models, genetically modified mouse lines with pericyte-specific deletions or overexpression of certain genes have been used to study the molecular mechanisms of pericyte function in the inner ear [22]. The Pdgfrb-CreERT2;Tgfbr2fl/fl mouse line, which has a pericyte-specific deletion of TGF-β receptor 2, has been used to study the role of TGF-β signaling in pericyte function and vascular stability in the inner ear [250,251,252]. Similarly, the Pdgfrb-CreERT2;Hif1afl/fl mouse line, which has a pericyte-specific deletion of HIF-1α, has been used to study the role of hypoxia signaling in pericyte function and angiogenesis [252]. 

### 7.2. Zebrafish Models

Zebrafish models have emerged as a valuable alternative to mouse models for studying pericytes in inner ear disorders due to their rapid development, high fecundity, and optical transparency [253,254,255]. Zebrafish have a well-characterized inner ear that shares many structural and functional similarities with the human inner ear, including the presence of sensory hair cells and supporting cells [243,256,257]. Moreover, the vascular anatomy of the zebrafish inner ear is highly conserved, with a network of capillaries and pericytes that closely resemble those found in mammals [258]. One advantage of using zebrafish models for studying pericytes in inner ear disorders is the ability to perform high-throughput genetic and pharmacological screens [259]. Zebrafish embryos can be easily manipulated genetically using morpholino antisense oligonucleotides or CRISPR-Cas9 technology, and can be exposed to various drugs or compounds in a 96-well format [260,261,262]. This allows for the rapid identification of genes or pathways that regulate pericyte function in the inner ear, as well as the discovery of potential therapeutic agents that can protect or regenerate pericytes [263]. Transgenic zebrafish lines have been developed to study pericytes in the inner ear, such as the Tg(pdgfrb:egfp) line, which expresses GFP under the control of the pdgfrb promoter and labels pericytes in the inner ear and other tissues [264,265]. 

Using this line, researchers have shown that pericytes are closely associated with the capillaries of the inner ear and that their distribution and morphology change during development and in response to injury [264]. Another transgenic line, the Tg(pdgfrb:mCherry;fli1a:egfp) line, which labels pericytes in red and endothelial cells in green, has been used to study the interactions between pericytes and endothelial cells in the inner ear and to visualize the effects of pericyte loss or dysfunction on vascular integrity [266].

### 7.3. In Vitro Models

**Figure 6:**
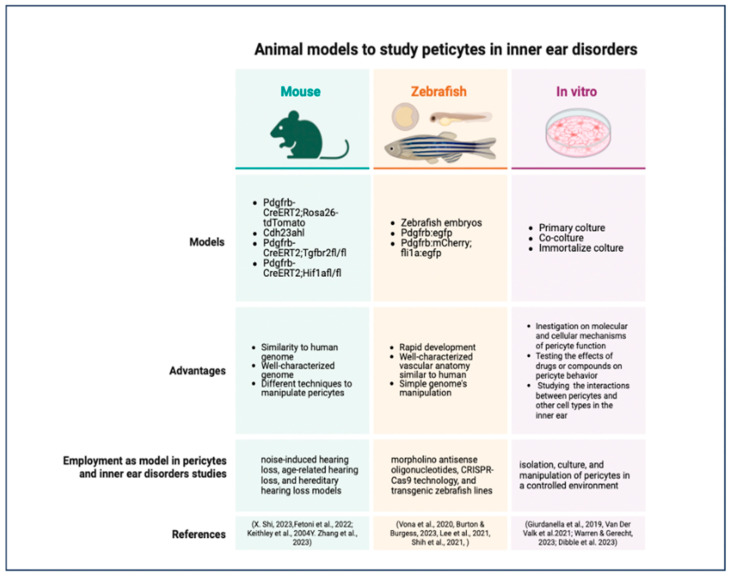
Schematic resume of the most commonly used animal model to study pericytes in inner ear disorders [22,44,55,247,255,259,262,265,267,269,270,279].

In vitro models are a valuable tool for studying pericytes in inner ear disorders, as they allow for the isolation, culture, and manipulation of pericytes in a controlled environment [267]. In vitro models can be used to study the molecular and cellular mechanisms of pericyte function, to test the effects of drugs or compounds on pericyte behavior, and to investigate the interactions between pericytes and other cell types in the inner ear [268,269]. One commonly used in vitro model for studying pericytes in the inner ear is the primary culture of pericytes isolated from the cochlear or vestibular microvasculature [270]. These cultures can be obtained by enzymatic digestion of the inner ear tissues and purification of pericytes based on their expression of specific markers, such as PDGFRβ, NG2, and αSMA [271]. Primary pericyte cultures can be used to study the proliferation, migration, and differentiation of pericytes in response to various stimuli, such as growth factors, cytokines, and hypoxia [272,273,274]. They can also be used to investigate the secretory profile of pericytes and their effects on other cell types, such as endothelial cells or neurons. In addition, the co-culture of pericytes with other cell types, such as endothelial cells, neurons, and sensory hair cells, represents a potential application [275,276,277]. These co-cultures can be used to study the bidirectional interactions between pericytes and other cells in the inner ear and to investigate the role of pericytes in regulating vascular permeability, neurovascular coupling, and hair cell survival [278]. Co-cultures of pericytes and endothelial cells have been used to study the effects of pericyte-derived factors on endothelial barrier function and to investigate the mechanisms of pericyte-mediated vascular stabilization in the inner ear [15,279]. In addition to primary cultures and co-cultures, immortalized pericyte cell lines have been developed from various tissues, including the brain and the retina [280,281,282]. These cell lines can be used as a more convenient and reproducible alternative to primary cultures and can be genetically modified to express or delete specific genes of interest [283,284,285] (Figure 6).

## 8. Current Research and Future Directions

The field of pericyte biology in the inner ear is rapidly evolving, with new discoveries and therapeutic approaches emerging at a fast pace [11]. 

Current research is focused on elucidating the molecular mechanisms of pericyte dysfunction in inner ear disorders, identifying pericyte-based diagnostic markers and therapeutic targets, and developing pericyte-based strategies for regenerative medicine in the inner ear [286].

### 8.1. Pericyte-Based Diagnostic Markers for Inner Ear Disorders

One of the major challenges in the diagnosis and treatment of inner ear disorders is the lack of specific and sensitive biomarkers that can detect the early stages of the disease and monitor the response to therapy [287]. 

Pericytes, as key regulators of vascular function and homeostasis in the inner ear, have emerged as a potential cellular marker for the diagnosis of various inner ear disorders, such as noise-induced hearing loss, age-related hearing loss, and Meniere’s disease [14,44]. 

Recent studies have identified several pericyte-derived factors that are altered in the blood or inner ear fluids of patients with inner ear disorders, and that may serve as diagnostic markers for these conditions [22]. The levels of angiopoietin-1, a pericyte derived factor that regulates endothelial cell survival and vascular stability, were significantly reduced in the serum of patients with pulmonary hypertension (PH), and the levels of angiopoietin-1 correlated with the severity of hearing loss and the response to treatment [288]. Similarly, a study by Zou et al. (2022) found that the levels of PDGF-BB, a growth factor that regulates pericyte recruitment and proliferation, were significantly increased in the perilymph of patients with Meniere’s disease, and that the levels of PDGF-BB correlated with the frequency and duration of vertigo attacks [289,290]. 

Supported by recent bibliographical research and bioinformatic analysis, it has been possible to classify the physiopathological biomarkers of the inner ear. Methodologically, the biomarkers identified have been categorized on the basis of their clinical, pathological, diagnostic, and predictive applications. From a molecular point of view, biomarkers are categorized as follows: inner ear-specific protein biomarkers that can be detected in peripheral blood, plasma, and serum, which include protein biomarkers (Otolin-1, Prestin, Matrilin-1), biomarkers of inflammation (IL-6, TNF-α, IL-1β), vasopressin, and BDNF; inner-ear-specific biomarkers detected in the perilymph or inner ear structures, which include Cochlin and Heat Shock Protein (HSP) and biomarkers of oxidative stress damage. Moreover, functional biomarkers that should be a metric or an indicator of a physiological disease have also been considered and categorized: elelctrovestibulography (EVestG), brainstem auditory evoked responses (ABR), and others. These findings suggest that pericyte-derived factors may serve as sensitive and specific biomarkers for inner ear disorders, and that their measurement in the blood or inner ear fluids may help to diagnose these conditions, predict their progression, and monitor the response to therapy [291,292] (Table 1). 

### 8.2. Pericyte Regeneration and Replacement Strategies

A promising approach to treating inner ear disorders is the regeneration or replacement of damaged or lost pericytes in order to restore vascular function and promote tissue repair [327,328,329,330]. Several strategies have been proposed for pericyte regeneration and replacement in the inner ear, including the use of stem cells, growth factors, and gene therapy [331,332].

One strategy for pericyte regeneration is the use of mesenchymal stem cells (MSCs), which have the ability to differentiate into pericytes and other vascular cell types, and to secrete pro-angiogenic and anti-inflammatory factors [219,220,333,334]. The transplantation of bone marrow-derived MSCs into the cochlea of mice with noise-induced hearing loss led to a significant increase in pericyte coverage and vascular density, and a significant improvement in hearing function, compared to untreated controls [223,335]. Similarly, the transplantation of adipose-derived MSCs into the cochlea of mice with age-related hearing loss led to a significant increase in pericyte number and a significant reduction in oxidative stress and inflammation, compared to untreated controls [336,337,338]. 

Another strategy for pericyte regeneration is the use of growth factors that promote pericyte recruitment, proliferation, and survival, such as PDGF-BB, TGF-β1, and VEGF [339]. A study by Hou et al. found that the local delivery of PDGF-BB into the cochlea of mice with noise-induced hearing loss led to a significant increase in pericyte coverage and a significant improvement in hearing function, compared to untreated controls [340]. Similarly, a study by Kawamoto et al. showed that the local delivery of TGF-β1 into the cochlea of mice with age-related hearing loss led to a significant increase in pericyte number and a significant reduction in oxidative stress and inflammation, compared to untreated controls [341,342]. 

Gene therapy is another promising approach to pericyte regeneration and replacement in the inner ear [225,343]. 

### 8.3. Drug Delivery-Targeting Pericytes

Pericytes, as key regulators of vascular function and homeostasis in the inner ear, are also emerging as promising targets for drug delivery in inner ear disorders [344]. By selectively delivering drugs to pericytes, it may be possible to modulate their function, prevent their dysfunction, and promote their regeneration without affecting other cell types in the inner ear [345]. Several strategies have been proposed for drug delivery-targeting pericytes in the inner ear, including the use of nanoparticles, liposomes, and exosomes [346,347]. 

These carriers can be engineered to express pericyte-specific ligands or antibodies on their surface, allowing them to bind to and be internalized by pericytes, while avoiding other cell types [348]. A study by Park et al. found that the delivery of dexamethasone-loaded nanoparticles conjugated with a PDGFRβ antibody into the cochlea of mice with noise-induced hearing loss led to a significant increase in pericyte coverage and a significant improvement in hearing function, compared to non-targeted nanoparticles or free dexamethasone [349]. 

Exosomes, which are small extracellular vesicles secreted by various cell types, have also been explored as carriers for drug delivery-targeting pericytes in the inner ear [350]. 

The delivery of exosomes derived from MSCs overexpressing PDGF-BB into the cochlea of mice with noise-induced hearing loss led to a significant increase in pericyte coverage and a significant improvement in hearing function, compared to untreated controls or exosomes derived from wild-type MSCs [351].

### 8.4. Pericyte-Based Approaches to Regenerative Medicine in the Inner Ear

Pericytes, as multipotent progenitor cells that can differentiate into various cell types, including vascular cells, neural cells, and mesenchymal cells, are also emerging as promising tools for regenerative medicine in the inner ear [352,353]. By exploiting the regenerative potential of pericytes, it may be possible to promote the repair and regeneration of damaged tissues in the inner ear, such as sensory hair cells, spiral ganglion neurons, and stria vascularis [354]. Several approaches have been proposed for using pericytes in regenerative medicine in the inner ear, including the use of pericyte-derived conditioned media, pericyte-derived extracellular vesicles, and pericyte-based tissue engineering [355,356,357]. The authors suggested that pericyte-derived conditioned media may contain pro-survival and pro-regenerative factors that can promote hair cell repair and regeneration.

The local delivery of extracellular vesicles derived from cochlear pericytes into the cochlea of mice with age-related hearing loss led to a significant increase in spiral ganglion neuron survival and a significant improvement in hearing function, compared to untreated controls or extracellular vesicles derived from other cell types [351]. The authors suggested that pericyte-derived extracellular vesicles may contain neurotrophic and pro-regenerative factors that can promote spiral ganglion neuron repair and regeneration. Pericyte-based tissue engineering is another promising approach to regenerative medicine in the inner ear [210]. The co-culture of cochlear pericytes with induced pluripotent stem cell-derived hair cell progenitors on a 3D collagen scaffold led to the formation of a functional sensory epithelium, with hair cell-like cells expressing functional mechanotransduction channels and synaptic connections with spiral ganglion neuron-like cells [358,359]. The authors suggested that pericyte-based tissue engineering may provide a promising platform for the generation of functional hair cell-like cells for cell replacement therapy in the inner ear. 

As the field of pericyte biology in the inner ear continues to advance, it will be important to integrate the knowledge gained from basic science research with the development of novel diagnostic and therapeutic strategies in order to translate these findings into clinical practice and improve the outcomes for patients with inner ear disorders. 

This will require a multidisciplinary approach involving collaborations between basic scientists, clinicians, and engineers, and the use of cutting-edge technologies, such as single-cell genomics, imaging, and biomaterials. With the rapid progress being made in this field, it is likely that pericyte-based approaches will play an increasingly important role in the diagnosis and treatment of inner ear disorders in the future. 

## 9. Conclusions

Pericytes are essential for maintaining the integrity and function of the inner ear vasculature, and their dysfunction has been implicated in various inner ear disorders. 

This review discusses the current understanding of pericyte biology in the inner ear, the mechanisms of pericyte dysfunction in inner ear disorders, and the potential of pericyte-based approaches for diagnosis and treatment. 

Key findings highlight the role of pericytes in regulating vascular permeability, blood flow, and angiogenesis in the inner ear. 

Pericyte dysfunction, characterized by loss, detachment, or phenotypic changes, involves oxidative stress, inflammation, and dysregulation of signaling pathways. Pericyte-derived factors, such as angiopoietin-1 and PDGF-BB, are potential biomarkers for inner ear disorders. 

Pericyte regeneration and replacement strategies, targeted drug delivery systems, and pericyte-based approaches to regenerative medicine seem to be promising in vascular repair and improving hearing function. 

Understanding pericyte dysfunction in inner ear disorders could provide new insights into pathophysiology, identify novel diagnostic markers and therapeutic targets, and lead to more effective and targeted therapies. Future research should focus on elucidating the molecular mechanisms of pericyte dysfunction, optimizing pericyte-based strategies, and exploring their potential for regenerative medicine. Multidisciplinary collaborations will be essential to translate basic science findings into clinical practice.

## Figures and Tables

**Table 1:** The main biomarkers used to diagnose inner ear disorders.

Molecular Biomarkers	Biomarker	Detection	Specificity	Reference
Inner Ear Specific Protein	Otolin-1		Inner ear hair cells	[291,293,294,295]
	Prestin	Blood, Serum, Plasma	Cochlear outer hair cells	[296,297]
	Matrilin-1		Upper airway cartilage	[292,298,299]
Inner Ear Inflammatory Protein	IL-6 TNF-α IL-1β Vasopressin BDNF	Blood, Serum, Plasma	Damage	[300,301,302] [291,292,303] [292,302] [88,304,305,306,307,308,309] [310,311,312,313]
Inner Ear Structure	Cochlin HPS-70 ROS	Perilymph	Cochlear and Vestibular Folding protein Stress Damage	[314,315,316] [317,318,319,320] [321,322,323]
Clinical Inner Ear Tests	Electrovestibulography Brainstem auditory evoked responses	Auditorysystems	Indicator of diseases	[291,292,310,324,325,326]

## References

[1] Smouha E. (2013). Inner Ear Disorders. NeuroRehabilitation.

[2] Young A.S., Rosengren S.M., Welgampola M.S., Day B.L., Lord S.R. (2018). Chapter 25—Disorders of the Inner-Ear Balance Organs and Their Pathways. Handbook of Clinical Neurology.

[3] Figtree W.V.C., Menant J.C., Chau A.T., Hübner P.P., Lord S.R., Migliaccio A.A. (2021). Prevalence of Vestibular Disorders in Independent People Over 50 That Experience Dizziness. Front. Neurol..

[4] Handa P.R., Kuhn A.M.B., Cunha F., Schaffleln R., Ganança F.F. (2005). Quality of Life in Patients with Benign Paroxysmal Positional Vertigo and/or Ménière’s Disease. Braz. J. Otorhinolaryngol..

[5] Azaiez H., Booth K.T., Ephraim S.S., Crone B., Black-Ziegelbein E.A., Marini R.J., Shearer A.E., Sloan-Heggen C.M., Kolbe D., Casavant T. (2018). Genomic Landscape and Mutational Signatures of Deafness-Associated Genes. Am. J. Hum. Genet..

[6] Kohrman D.C., Wan G., Cassinotti L., Corfas G. (2020). Hidden Hearing Loss: A Disorder with Multiple Etiologies and Mechanisms. Cold Spring Harb. Perspect. Med..

[7] Bachor E., Selig Y.K., Jahnke K., Rettinger G., Karmody C.S. (2001). Vascular Variations of the Inner Ear. Acta Oto-Laryngol..

[8] Le Prell C.G., Clavier O.H., Bao J. (2023). Noise-Induced Hearing Disorders: Clinical and Investigational Tools. J. Acoust. Soc. Am..

[9] Ishiyama G., Lopez I.A., Acuna D., Ishiyama A. (2019). Investigations of the Microvasculature of the Human Macula Utricle in Meniere’s Disease. Front. Cell. Neurosci..

[10] Alarcon-Martinez L., Yemisci M., Dalkara T. (2021). Pericyte Morphology and Function. Histol. Histopathol..

[11] Dessalles C.A., Babataheri A., Barakat A.I. (2021). Pericyte Mechanics and Mechanobiology. J. Cell Sci..

[12] Van Dijk C.G.M., Nieuweboer F.E., Pei J.Y., Xu Y.J., Burgisser P., Van Mulligen E., El Azzouzi H., Duncker D.J., Verhaar M.C., Cheng C. (2015). The Complex Mural Cell: Pericyte Function in Health and Disease. Int. J. Cardiol..

[13] Sims D.E. (2000). Diversity Within Pericytes. Clin. Exp. Pharmacol. Physiol..

[14] Peppiatt C.M., Howarth C., Mobbs P., Attwell D. (2006). Bidirectional Control of CNS Capillary Diameter by Pericytes. Nature.

[15] Neng L., Zhang F., Kachelmeier A., Shi X. (2013). Endothelial Cell, Pericyte, and Perivascular Resident Macrophage-Type Melanocyte Interactions Regulate Cochlear Intrastrial Fluid–Blood Barrier Permeability. J. Assoc. Res. Otolaryngol..

[16] Zhang J., Chen S., Cai J., Hou Z., Wang X., Kachelmeier A., Shi X. (2017). Culture Media-Based Selection of Endothelial Cells, Pericytes, and Perivascular-Resident Macrophage-like Melanocytes from the Young Mouse Vestibular System. Hear. Res..

[17] Ishiyama G., Lopez I.A., Ishiyama P., Vinters H.V., Ishiyama A. (2017). The Blood Labyrinthine Barrier in the Human Normal and Meniere’s Disease Macula Utricle. Sci. Rep..

[18] Gerhardt H., Betsholtz C. (2003). Endothelial-Pericyte Interactions in Angiogenesis. Cell Tissue Res..

[19] Matsuki M., Kabara M., Saito Y., Shimamura K., Minoshima A., Nishimura M., Aonuma T., Takehara N., Hasebe N., Kawabe J. (2015). Ninjurin1 Is a Novel Factor to Regulate Angiogenesis Through the Function of Pericytes. Circ. J..

[20] Teichert M., Milde L., Holm A., Stanicek L., Gengenbacher N., Savant S., Ruckdeschel T., Hasanov Z., Srivastava K., Hu J. (2017). Pericyte-Expressed Tie2 Controls Angiogenesis and Vessel Maturation. Nat. Commun..

[21] Sharma K., Zhang Y., Paudel K.R., Kachelmeier A., Hansbro P.M., Shi X. (2022). The Emerging Role of Pericyte-Derived Extracellular Vesicles in Vascular and Neurological Health. Cells.

[22] Zhang Y., Neng L., Sharma K., Hou Z., Johnson A., Song J., Dabdoub A., Shi X. (2023). Pericytes Control Vascular Stability and Auditory Spiral Ganglion Neuron Survival. eLife.

[23] Laredo F., Plebanski J., Tedeschi A. (2019). Pericytes: Problems and Promises for CNS Repair. Front. Cell. Neurosci..

[24] Staniszewska M., Gu X., Romano C., Kazlauskas A. (2012). A Phage Display-Based Approach to Investigate Abnormal Neovessels of the Retina. Invest. Ophthalmol. Vis. Sci..

[25] Giurdanella G., Anfuso C.D., Olivieri M., Lupo G., Caporarello N., Eandi C.M., Drago F., Bucolo C., Salomone S. (2015). Aflibercept, Bevacizumab and Ranibizumab Prevent Glucose-Induced Damage in Human Retinal Pericytes in Vitro, through a PLA2/COX-2/VEGF-A Pathway. Biochem. Pharmacol..

[26] Bryant D., Pauzuolyte V., Ingham N.J., Patel A., Pagarkar W., Anderson L.A., Smith K.E., Moulding D.A., Leong Y.C., Jafree D.J. (2022). The Timing of Auditory Sensory Deficits in Norrie Disease Has Implications for Therapeutic Intervention. JCI Insight.

[27] Dufek B., Meehan D.T., Delimont D., Samuelson G., Madison J., Shi X., Boettcher F., Trosky V., Gratton M.A., Cosgrove D. (2020). Pericyte Abnormalities Precede Strial Capillary Basement Membrane Thickening in Alport Mice. Hear. Res..

[28] Hou Z., Neng L., Zhang J., Cai J., Wang X., Zhang Y., Lopez I.A., Shi X. (2020). Acoustic Trauma Causes Cochlear Pericyte-to-Myofibroblast-like Cell Transformation and Vascular Degeneration, and Transplantation of New Pericytes Prevents Vascular Atrophy. Am. J. Pathol..

[29] Bergers G., Song S. (2005). The Role of Pericytes in Blood-Vessel Formation and Maintenance. Neuro-Oncology.

[30] Betsholtz C., Lindblom P., Gerhardt H. (2005). Role of Pericytes in Vascular Morphogenesis. Mechanisms of Angiogenesis.

[31] Kutcher M.E., Herman I.M. (2009). The Pericyte: Cellular Regulator of Microvascular Blood Flow. Microvasc. Res..

[32] Matsunaga T., Kanzaki J., Hosoda Y. (1996). The Vasculature of the Peripheral Portion of the Human Eighth Cranial Nerve. Hear. Res..

[33] Canis M., Bertlich M. (2019). Cochlear Capillary Pericytes. Adv. Exp. Med. Biol..

[34] Zhang Z.-S., Zhou H.-N., He S.-S., Xue M.-Y., Li T., Liu L.-M. (2020). Research Advances in Pericyte Function and Their Roles in Diseases. Chin. J. Traumatol..

[35] Jiang Y., Yao H., Chen J., Zhang J., Rao Y., Chen K., Tang Y. (2020). The distribution of perivascular-resident cells in blood-labyrinth barrier observed with two-photon fluorescence microscope and Imaris deconvolution. J. Clin. Otorhinolaryngol. Head Neck Surg..

[36] Longden T.A., Zhao G., Hariharan A., Lederer W.J. (2023). Pericytes and the Control of Blood Flow in Brain and Heart. Annu. Rev. Physiol..

[37] Zheng Z., Chopp M., Chen J. (2020). Multifaceted Roles of Pericytes in Central Nervous System Homeostasis and Disease. J. Cereb. Blood Flow. Metab..

[38] Zhang J., Hou Z., Wang X., Jiang H., Neng L., Zhang Y., Yu Q., Burwood G., Song J., Auer M. (2021). VEGFA165 Gene Therapy Ameliorates Blood-Labyrinth Barrier Breakdown and Hearing Loss. JCI Insight.

[39] Ernfors P., Van De Water T., Loring J., Jaenisch R. (1995). Complementary Roles of BDNF and NT-3 in Vestibular and Auditory Development. Neuron.

[40] Singer W., Panford-Walsh R., Knipper M. (2014). The Function of BDNF in the Adult Auditory System. Neuropharmacology.

[41] Jiang H., Wang X., Zhang J., Kachelmeier A., Lopez I.A., Shi X. (2019). Microvascular Networks in the Area of the Auditory Peripheral Nervous System. Hear. Res..

[42] Gaceb A., Özen I., Padel T., Barbariga M., Paul G. (2018). Pericytes Secrete Pro-Regenerative Molecules in Response to Platelet-Derived Growth Factor-BB. J. Cereb. Blood Flow. Metab..

[43] Zhang F., Zhang J., Neng L., Shi X. (2013). Characterization and Inflammatory Response of Perivascular-Resident Macrophage-like Melanocytes in the Vestibular System. J. Assoc. Res. Otolaryngol..

[44] Shi X. (2023). Research Advances in Cochlear Pericytes and Hearing Loss. Hear. Res..

[45] Neng L., Zhang J., Yang J., Zhang F., Lopez I.A., Dong M., Shi X. (2015). Structural Changes in Thestrial Blood-Labyrinth Barrier of Aged C57BL/6 Mice. Cell Tissue Res..

[46] Sekulic M., Puche R., Bodmer D., Petkovic V. (2023). Human Blood-Labyrinth Barrier Model to Study the Effects of Cytokines and Inflammation. Front. Mol. Neurosci..

[47] Choi Y.-K., Kim K.-W. (2008). Blood-Neural Barrier: Its Diversity and Coordinated Cell-to-Cell Communication. BMB Rep..

[48] Díaz-Flores L., Gutiérrez R., García M.P., González-Gómez M., Díaz-Flores L., Carrasco J.L., Madrid J.F., Rodríguez Bello A. (2022). Comparison of the Behavior of Perivascular Cells (Pericytes and CD34+ Stromal Cell/Telocytes) in Sprouting and Intussusceptive Angiogenesis. Int. J. Mol. Sci..

[49] Díaz-Flores L., Gutiérrez R., Madrid J.F., Varela H., Valladares F., Acosta E., Martín-Vasallo P., Díaz-Flores L. (2009). Pericytes. Morphofunction, Interactions and Pathology in a Quiescent and Activated Mesenchymal Cell Niche. Histol. Histopathol..

[50] Ribatti D., Nico B., Crivellato E. (2011). The Role of Pericytes in Angiogenesis. Int. J. Dev. Biol..

[51] Fisher R.A., Miners J.S., Love S. (2022). Pathological Changes within the Cerebral Vasculature in Alzheimer’s Disease: New Perspectives. Brain Pathol..

[52] Shi H., Koronyo Y., Rentsendorj A., Regis G.C., Sheyn J., Fuchs D.-T., Kramerov A.A., Ljubimov A.V., Dumitrascu O.M., Rodriguez A.R. (2020). Identification of Early Pericyte Loss and Vascular Amyloidosis in Alzheimer’s Disease Retina. Acta Neuropathol..

[53] Zlokovic B.V. (2011). Neurovascular Pathways to Neurodegeneration in Alzheimer’s Disease and Other Disorders. Nat. Rev. Neurosci..

[54] Cosentino A., Agafonova A., Modafferi S., Trovato Salinaro A., Scuto M., Maiolino L., Fritsch T., Calabrese E.J., Lupo G., Anfuso C.D. (2024). Blood–Labyrinth Barrier in Health and Diseases: Effect of Hormetic Nutrients. Antioxid. Redox Signal..

[55] Fetoni A.R., Pisani A., Rolesi R., Paciello F., Viziano A., Moleti A., Sisto R., Troiani D., Paludetti G., Grassi C. (2022). Early Noise-Induced Hearing Loss Accelerates Presbycusis Altering Aging Processes in the Cochlea. Front. Aging Neurosci..

[56] Zhou Y., Lu H., Tan C.Y., Qu Z.W., Chang Y.C., Han Z.W., Si J.Q., Ma K.T., Li L. (2019). Changes of BK(Ca) on vascular striaepericytes of D-galactose-induced aging model in guinea pigs. Chin. J. Otorhinolaryngol. Head Neck Surg..

[57] Thulasiram M.R., Ogier J.M., Dabdoub A. (2022). Hearing Function, Degeneration, and Disease: Spotlight on the Stria Vascularis. Front. Cell Dev. Biol..

[58] Bhattacharya A., Kaushik D.K., Lozinski B.M., Yong V.W. (2020). Beyond Barrier Functions: Roles of Pericytes in Homeostasis and Regulation of Neuroinflammation. J. Neurosci. Res..

[59] Hung C.F., Holton S., Chow Y.-H., Liles W.C., Gharib S.A., Altemeier W.A. (2021). Pericyte-like Cells Undergo Transcriptional Reprogramming and Distinct Functional Adaptations in Acute Lung Injury. FASEB J..

[60] Alex L., Tuleta I., Hernandez S.C., Hanna A., Venugopal H., Astorkia M., Humeres C., Kubota A., Su K., Zheng D. (2023). Cardiac Pericytes Acquire a Fibrogenic Phenotype and Contribute to Vascular Maturation After Myocardial Infarction. Circulation.

[61] Dulmovits B.M., Herman I.M. (2012). Microvascular Remodeling and Wound Healing: A Role for Pericytes. Int. J. Biochem. Cell Biol..

[62] Gopinathan G., Milagre C., Pearce O.M.T., Reynolds L.E., Hodivala-Dilke K., Leinster D.A., Zhong H., Hollingsworth R.E., Thompson R., Whiteford J.R. (2015). Interleukin-6 Stimulates Defective Angiogenesis. Cancer Res..

[63] Yao Y., Tsirka S.E. (2014). Monocyte Chemoattractant Protein-1 and the Blood–Brain Barrier. Cell. Mol. Life Sci..

[64] Moriguchi M., Masutani H., Sugita M., Matsunaga K., Okamoto J., Nakai Y. (1991). Development of Inner Ear Vessels. A Scanning Electron Microscopic Study. Acta Otolaryngol. Suppl..

[65] Balogová Z., Popelář J., Chiumenti F., Chumak T., Burianová J.S., Rybalko N., Syka J. (2017). Age-Related Differences in Hearing Function and Cochlear Morphology between Male and Female Fischer 344 Rats. Front. Aging Neurosci..

[66] Thomopoulos G.N., Spicer S.S., Gratton M.A., Schulte B.A. (1997). Age-Related Thickening of Basement Membrane in Stria Vascularis Capillaries. Hear. Res..

[67] Ghelfi E., Grondin Y., Millet E.J., Bartos A., Bortoni M., Oliveira Gomes Dos Santos C., Trevino-Villarreal H.J., Sepulveda R., Rogers R. (2018). In Vitro Gentamicin Exposure Alters Caveolae Protein Profile in Cochlear Spiral Ligament Pericytes. Proteome Sci..

[68] Zhang N., Cai J., Xu L., Wang H., Liu W. (2020). Cisplatin-Induced Stria Vascularis Damage Is Associated with Inflammation and Fibrosis. Neural Plast..

[69] Beltramo E., Porta M. (2013). Pericyte Loss in Diabetic Retinopathy: Mechanisms and Consequences. Curr. Med. Chem..

[70] Winkler E.A., Sengillo J.D., Bell R.D., Wang J., Zlokovic B.V. (2012). Blood-Spinal Cord Barrier Pericyte Reductions Contribute to Increased Capillary Permeability. J. Cereb. Blood Flow. Metab..

[71] Shi X. (2009). Cochlear Pericyte Responses to Acoustic Trauma and the Involvement of Hypoxia-Inducible Factor-1alpha and Vascular Endothelial Growth Factor. Am. J. Pathol..

[72] Koch K., Lindner M., Fleck A.-K., Liebmann M., Eschborn M., Zondler L., Diéguez-Hurtado R., Adams R.H., Meyer Zu Hörste G., Zarbock A. (2022). CNS Pericytes Modulate Local T Cell Infiltration in EAE. Int. J. Mol. Sci..

[73] van Hezel M.E., Nieuwland R., van Bruggen R., Juffermans N.P. (2017). The Ability of Extracellular Vesicles to Induce a Pro-Inflammatory Host Response. Int. J. Mol. Sci..

[74] Shearer A.E., Hildebrand M.S., Schaefer A.M., Smith R.J., Adam M.P., Feldman J., Mirzaa G.M., Pagon R.A., Wallace S.E., Bean L.J., Gripp K.W., Amemiya A. (1993). Genetic Hearing Loss Overview. GeneReviews^®^.

[75] Young A., Ng M. (2024). Genetic Hearing Loss. StatPearls.

[76] Yu W., Zong S., Du P., Zhou P., Li H., Wang E., Xiao H. (2021). Role of the Stria Vascularis in the Pathogenesis of Sensorineural Hearing Loss: A Narrative Review. Front. Neurosci..

[77] Ohlmann A., Tamm E.R. (2012). Norrin: Molecular and Functional Properties of an Angiogenic and Neuroprotective Growth Factor. Prog. Retin. Eye Res..

[78] Zuercher J., Fritzsche M., Feil S., Mohn L., Berger W. (2012). Norrin Stimulates Cell Proliferation in the Superficial Retinal Vascular Plexus and Is Pivotal for the Recruitment of Mural Cells. Hum. Mol. Genet..

[79] Hayashi Y., Chiang H., Tian C., Indzhykulian A.A., Edge A.S.B. (2021). Norrie Disease Protein Is Essential for Cochlear Hair Cell Maturation. Proc. Natl. Acad. Sci. USA.

[80] Nyegaard M., Rendtorff N.D., Nielsen M.S., Corydon T.J., Demontis D., Starnawska A., Hedemand A., Buniello A., Niola F., Overgaard M.T. (2015). A Novel Locus Harbouring a Functional CD164 Nonsense Mutation Identified in a Large Danish Family with Nonsyndromic Hearing Impairment. PLoS Genet..

[81] Winkler E.A., Bell R.D., Zlokovic B.V. (2010). Pericyte-Specific Expression of PDGF Beta Receptor in Mouse Models with Normal and Deficient PDGF Beta Receptor Signaling. Mol. Neurodegener..

[82] Strupp M., Brandt T., Dieterich M. (2023). Acute Unilateral Vestibulopathy/Vestibular Neuritis. Vertigo and Dizziness: Common Complaints.

[83] Da Costa S.S., De Sousa L.C.A., De Toledo Piza M.R. (2002). Meniere’s Disease: Overview, Epidemiology, and Natural History. Otolaryngol. Clin. N. Am..

[84] Gluth M.B. (2020). On the Relationship Between Menière’s Disease and Endolymphatic Hydrops. Otol. Neurotol..

[85] Guajardo-Vergara C., Suárez-Vega V., Dominguez P., Manrique-Huarte R., Arbizu L., Pérez-Fernández N. (2022). Endolymphatic Hydrops in the Unaffected Ear of Patients with Unilateral Ménière’s Disease. Eur. Arch. Otorhinolaryngol..

[86] Gürkov R., Pyykö I., Zou J., Kentala E. (2016). What Is Menière’s Disease? A Contemporary Re-Evaluation of Endolymphatic Hydrops. J. Neurol..

[87] Ishiyama G., Wester J., Lopez I.A., Beltran-Parrazal L., Ishiyama A. (2018). Oxidative Stress in the Blood Labyrinthine Barrier in the Macula Utricle of Meniere’s Disease Patients. Front. Physiol..

[88] Salt A.N., Plontke S.K. (2010). Endolymphatic Hydrops: Pathophysiology and Experimental Models. Otolaryngol. Clin. N. Am..

[89] Perez-Carpena P., Lopez-Escamez J.A. (2020). Current Understanding and Clinical Management of Meniere’s Disease: A Systematic Review. Semin. Neurol..

[90] Haeussler S.M., Zabaneh S.I., Stegemann M., Olze H., Böttcher A., Stölzel K. (2022). Is Vestibular Neuropathy Rather a Neuritis?. Cureus.

[91] Naranjo O., Torices S., Clifford P.R., Daftari M.T., Osborne O.M., Fattakhov N., Toborek M. (2022). Pericyte Infection by HIV-1: A Fatal Attraction. Retrovirology.

[92] Bartual-Pastor J. (2005). Vestibular Neuritis: Etiopathogenesis. Rev. Laryngol. Otol. Rhinol..

[93] Davis L.E. (1993). Viruses and Vestibular Neuritis: Review of Human and Animal Studies. Acta Oto-Laryngol..

[94] Hirata T., Sekitani T., Okinaka Y., Matsuda Y. (1989). Serovirological Study of Vestibular Neuronitis. Acta Oto-Laryngol..

[95] Zhao Z., Liu X., Zong Y., Shi X., Sun Y. (2023). Cellular Processes Induced by HSV-1 Infections in Vestibular Neuritis. Viruses.

[96] Aedo-Sánchez C., Gutiérrez G., Aguilar-Vidal E. (2024). COVID-19 and Vestibular Symptoms and Assessment: A Review. Audiol. Neurotol..

[97] Giannantonio S., Scorpecci A., Montemurri B., Marsella P. (2021). Case of COVID-19-Induced Vestibular Neuritis in a Child. BMJ Case Rep..

[98] Malayala S.V., Mohan G., Vasireddy D., Atluri P. (2021). A Case Series of Vestibular Symptoms in Positive or Suspected COVID-19 Patients. Infez. Med..

[99] Malayala S.V., Raza A. (2020). A Case of COVID-19-Induced Vestibular Neuritis. Cureus.

[100] Mat Q., Noël A., Loiselet L., Tainmont S., Chiesa-Estomba C.M., Lechien J.R., Duterme J.-P. (2023). Vestibular Neuritis as Clinical Presentation of COVID-19. Ear Nose Throat J..

[101] Kim H.-J., Park J., Kim J.-S. (2021). Update on Benign Paroxysmal Positional Vertigo. J. Neurol..

[102] You P., Instrum R., Parnes L. (2019). Benign Paroxysmal Positional Vertigo. Laryngoscope Investig. Oto.

[103] Walther L.E., Westhofen M. (2007). Presbyvertigo-Aging of Otoconia and Vestibular Sensory Cells. J. Vestib. Res..

[104] Parnes L.S., Agrawal S.K., Atlas J. (2003). Diagnosis and Management of Benign Paroxysmal Positional Vertigo (BPPV). CMAJ.

[105] Barozzi S., Socci M., Ginocchio D., Filipponi E., Martinazzoli M.G.T., Cesarani A. (2013). Benign Paroxysmal Positional Vertigo and Tinnitus. Int. Tinnitus J..

[106] Gavalas G.J., Passou E.M., Vathilakis J.M. (2001). Tinnitus of Vestibular Origin. Scand. Audiol..

[107] Kocabaş E., Kutluhan A., Müjdeci B. (2021). The Evaluation of Tinnitus and Auditory Brainstem Response in Benign Paroxysmal Positional Vertigo Accompanied by Tinnitus. Eur. Arch. Otorhinolaryngol..

[108] Langguth B., Kreuzer P.M., Kleinjung T., De Ridder D. (2013). Tinnitus: Causes and Clinical Management. Lancet Neurol..

[109] Feng J., Bendiske J., Morest D.K. (2012). Degeneration in the Ventral Cochlear Nucleus after Severe Noise Damage in Mice. J. Neurosci. Res..

[110] Mulders W.H.A.M., Vooys V., Makowiecki K., Tang A.D., Rodger J. (2016). The Effects of Repetitive Transcranial Magnetic Stimulation in an Animal Model of Tinnitus. Sci. Rep..

[111] Tan J., Rüttiger L., Panford-Walsh R., Singer W., Schulze H., Kilian S.B., Hadjab S., Zimmermann U., Köpschall I., Rohbock K. (2007). Tinnitus Behavior and Hearing Function Correlate with the Reciprocal Expression Patterns of BDNF and Arg3.1/Arc in Auditory Neurons Following Acoustic Trauma. Neuroscience.

[112] Ohlemiller K.K., Dwyer N., Henson V., Fasman K., Hirose K. (2024). A Critical Evaluation of “Leakage” at the Cochlear Blood-Stria-Barrier and Its Functional Significance. Front. Mol. Neurosci..

[113] De Siati R.D., Rosenzweig F., Gersdorff G., Gregoire A., Rombaux P., Deggouj N. (2020). Auditory Neuropathy Spectrum Disorders: From Diagnosis to Treatment: Literature Review and Case Reports. JCM.

[114] Jang M.W., Oh D.-Y., Yi E., Liu X., Ling J., Kim N., Sharma K., Kim T.Y., Lee S., Kim A.-R. (2021). A Nonsense *TMEM43* Variant Leads to Disruption of Connexin-Linked Function and Autosomal Dominant Auditory Neuropathy Spectrum Disorder. Proc. Natl. Acad. Sci. USA.

[115] Harris S.L., Kazmi Erczak M., Pangršič T., Shah P., Chuchvara N., Barrantes-Freer A., Moser T., Schwander M. (2017). Conditional Deletion of Pejvakin in Adult Outer Hair Cells Causes Progressive Hearing Loss in Mice. Neuroscience.

[116] Yuan Y., Shi F., Yin Y., Tong M., Lang H., Polley D.B., Liberman M.C., Edge A.S.B. (2014). Ouabain-Induced Cochlear Nerve Degeneration: Synaptic Loss and Plasticity in a Mouse Model of Auditory Neuropathy. J. Assoc. Res. Otolaryngol..

[117] Norrix L.W., Velenovsky D.S. (2014). Auditory Neuropathy Spectrum Disorder: A Review. J. Speech Lang. Hear. Res..

[118] Li P., Fan H. (2023). Pericyte Loss in Diseases. Cells.

[119] Nikolakopoulou A.M., Montagne A., Kisler K., Dai Z., Wang Y., Huuskonen M.T., Sagare A.P., Lazic D., Sweeney M.D., Kong P. (2019). Pericyte Loss Leads to Circulatory Failure and Pleiotrophin Depletion Causing Neuron Loss. Nat. Neurosci..

[120] Shi X. (2016). Pathophysiology of the Cochlear Intrastrial Fluid-Blood Barrier (Review). Hear. Res..

[121] Ishitsuka K., Ago T., Arimura K., Nakamura K., Tokami H., Makihara N., Kuroda J., Kamouchi M., Kitazono T. (2012). Neurotrophin Production in Brain Pericytes during Hypoxia: A Role of Pericytes for Neuroprotection. Microvasc. Res..

[122] Medina-Flores F., Hurtado-Alvarado G., Deli M.A., Gómez-González B. (2023). The Active Role of Pericytes During Neuroinflammation in the Adult Brain. Cell Mol. Neurobiol..

[123] Kaga K. (2016). Auditory Nerve Disease and Auditory Neuropathy Spectrum Disorders. Auris Nasus Larynx.

[124] Chen L., Deng H., Cui H., Fang J., Zuo Z., Deng J., Li Y., Wang X., Zhao L. (2018). Inflammatory Responses and Inflammation-Associated Diseases in Organs. Oncotarget.

[125] Marangio A., Biccari A., D’Angelo E., Sensi F., Spolverato G., Pucciarelli S., Agostini M. (2022). The Study of the Extracellular Matrix in Chronic Inflammation: A Way to Prevent Cancer Initiation?. Cancers.

[126] Barkwill D., Arora R. (2024). Labyrinthitis. StatPearls.

[127] Miwa T., Okano T. (2022). Role of Inner Ear Macrophages and Autoimmune/Autoinflammatory Mechanisms in the Pathophysiology of Inner Ear Disease. Front. Neurol..

[128] Rahman M.U., Poe D.S., Choi H.K. (2001). Autoimmune Vestibulo-Cochlear Disorders. Curr. Opin. Rheumatol..

[129] Dai Q., Long L., Zhao H., Wang R., Zheng H., Duan M. (2023). Genetic Advances in Meniere Disease. Mol. Biol. Rep..

[130] Li L., Chen D., Lin X., Luo J., Tan J., Li P. (2023). Understanding the Role of Inflammation in Sensorineural Hearing Loss: Current Goals and Future Prospects. Brain-X.

[131] Schrimpf C., Teebken O.E., Wilhelmi M., Duffield J.S. (2014). The Role of Pericyte Detachment in Vascular Rarefaction. J. Vasc. Res..

[132] Aranda-Rivera A.K., Cruz-Gregorio A., Arancibia-Hernández Y.L., Hernández-Cruz E.Y., Pedraza-Chaverri J. (2022). RONS and Oxidative Stress: An Overview of Basic Concepts. Oxygen.

[133] Pizzino G., Irrera N., Cucinotta M., Pallio G., Mannino F., Arcoraci V., Squadrito F., Altavilla D., Bitto A. (2017). Oxidative Stress: Harms and Benefits for Human Health. Oxidative Med. Cell. Longev..

[134] Kishimoto-Urata M., Urata S., Fujimoto C., Yamasoba T. (2022). Role of Oxidative Stress and Antioxidants in Acquired Inner Ear Disorders. Antioxidants.

[135] Shahab M., Jamesdaniel S. (2022). Nitrative Stress and Auditory Dysfunction. Pharmaceuticals.

[136] Tan W.J.T., Song L. (2023). Role of Mitochondrial Dysfunction and Oxidative Stress in Sensorineural Hearing Loss. Hear. Res..

[137] Armulik A., Genové G., Mäe M., Nisancioglu M.H., Wallgard E., Niaudet C., He L., Norlin J., Lindblom P., Strittmatter K. (2010). Pericytes Regulate the Blood–Brain Barrier. Nature.

[138] Carvalho C., Moreira P.I. (2018). Oxidative Stress: A Major Player in Cerebrovascular Alterations Associated to Neurodegenerative Events. Front. Physiol..

[139] Hayden M.R., Yang Y., Habibi J., Bagree S.V., Sowers J.R. (2010). Pericytopathy: Oxidative Stress and Impaired Cellular Longevity in the Pancreas and Skeletal Muscle in Metabolic Syndrome and Type 2 Diabetes. Oxidative Med. Cell. Longev..

[140] Staiculescu M., Foote C., Meininger G., Martinez-Lemus L. (2014). The Role of Reactive Oxygen Species in Microvascular Remodeling. Int. J. Mol. Sci..

[141] Ding X., Zhang M., Gu R., Xu G., Wu H. (2017). Activated Microglia Induce the Production of Reactive Oxygen Species and Promote Apoptosis of Co-Cultured Retinal Microvascular Pericytes. Graefes Arch. Clin. Exp. Ophthalmol..

[142] Shah G.N., Morofuji Y., Banks W.A., Price T.O. (2013). High Glucose-Induced Mitochondrial Respiration and Reactive Oxygen Species in Mouse Cerebral Pericytes Is Reversed by Pharmacological Inhibition of Mitochondrial Carbonic Anhydrases: Implications for Cerebral Microvascular Disease in Diabetes. Biochem. Biophys. Res. Commun..

[143] He P., Talukder M.A.H., Gao F. (2020). Oxidative Stress and Microvessel Barrier Dysfunction. Front. Physiol..

[144] Hill J., Rom S., Ramirez S.H., Persidsky Y. (2014). Emerging Roles of Pericytes in the Regulation of the Neurovascular Unit in Health and Disease. J. Neuroimmune Pharmacol..

[145] Navarro R., Compte M., Álvarez-Vallina L., Sanz L. (2016). Immune Regulation by Pericytes: Modulating Innate and Adaptive Immunity. Front. Immunol..

[146] Wong D., Dorovini-Zis K. (1992). Upregulation of Intercellular Adhesion Molecule-1 (ICAM-1) Expression in Primary Cultures of Human Brain Microvessel Endothelial Cells by Cytokines and Lipopolysaccharide. J. Neuroimmunol..

[147] Yang J., Ran M., Li H., Lin Y., Ma K., Yang Y., Fu X., Yang S. (2022). New Insight into Neurological Degeneration: Inflammatory Cytokines and Blood–Brain Barrier. Front. Mol. Neurosci..

[148] Yang S., Jin H., Zhu Y., Wan Y., Opoku E.N., Zhu L., Hu B. (2017). Diverse Functions and Mechanisms of Pericytes in Ischemic Stroke. Curr. Neuropharmacol..

[149] Rustenhoven J., Aalderink M., Scotter E.L., Oldfield R.L., Bergin P.S., Mee E.W., Graham E.S., Faull R.L.M., Curtis M.A., Park T.I.-H. (2016). TGF-Beta1 Regulates Human Brain Pericyte Inflammatory Processes Involved in Neurovasculature Function. J. Neuroinflam..

[150] Batlle E., Massagué J. (2019). Transforming Growth Factor-β Signaling in Immunity and Cancer. Immunity.

[151] Massagué J., Sheppard D. (2023). TGF-β Signaling in Health and Disease. Cell.

[152] Sanjabi S., Oh S.A., Li M.O. (2017). Regulation of the Immune Response by TGF-β: From Conception to Autoimmunity and Infection. Cold Spring Harb. Perspect. Biol..

[153] Kopke R., Staecker H., Lefebvre P., Malgrange B., Moonen G., Ruben R.J., Van De Water T.R. (1996). Effect of Neurotrophic Factors on the Inner Ear: Clinical Implications. Acta Oto-Laryngol..

[154] Fu J., Liang H., Yuan P., Wei Z., Zhong P. (2023). Brain Pericyte Biology: From Physiopathological Mechanisms to Potential Therapeutic Applications in Ischemic Stroke. Front. Cell. Neurosci..

[155] Hamilton N.B. (2010). Pericyte-Mediated Regulation of Capillary Diameter: A Component of Neurovascular Coupling in Health and Disease. Front. Neuroenerg..

[156] Nauta T., Van Hinsbergh V., Koolwijk P. (2014). Hypoxic Signaling During Tissue Repair and Regenerative Medicine. Int. J. Mol. Sci..

[157] Tsao C.-C., Baumann J., Huang S.-F., Kindler D., Schroeter A., Kachappilly N., Gassmann M., Rudin M., Ogunshola O.O. (2021). Pericyte Hypoxia-Inducible Factor-1 (HIF-1) Drives Blood-Brain Barrier Disruption and Impacts Acute Ischemic Stroke Outcome. Angiogenesis.

[158] Candelario-Jalil E., Dijkhuizen R.M., Magnus T. (2022). Neuroinflammation, Stroke, Blood-Brain Barrier Dysfunction, and Imaging Modalities. Stroke.

[159] Chappell J.C., Bautch V.L. (2010). Vascular Development. Current Topics in Developmental Biology.

[160] Sweeney M.D., Ayyadurai S., Zlokovic B.V. (2016). Pericytes of the Neurovascular Unit: Key Functions and Signaling Pathways. Nat. Neurosci..

[161] Battey J.F. (2000). A Genetic Approach to Understanding Inner Ear Function. J. Clin. Investig..

[162] Raviv D., Dror A.A., Avraham K.B. (2010). Hearing Loss: A Common Disorder Caused by Many Rare Alleles. Ann. N. Y. Acad. Sci..

[163] Heldin C.-H., Lennartsson J. (2013). Structural and Functional Properties of Platelet-Derived Growth Factor and Stem Cell Factor Receptors. Cold Spring Harb. Perspect. Biol..

[164] Hellström M., Kalén M., Lindahl P., Abramsson A., Betsholtz C. (1999). Role of PDGF-B and PDGFR-β in Recruitment of Vascular Smooth Muscle Cells and Pericytes during Embryonic Blood Vessel Formation in the Mouse. Development.

[165] Raica M., Cimpean A.M. (2010). Platelet-Derived Growth Factor (PDGF)/PDGF Receptors (PDGFR) Axis as Target for Antitumor and Antiangiogenic Therapy. Pharmaceuticals.

[166] Arts F.A., Velghe A.I., Stevens M., Renauld J., Essaghir A., Demoulin J. (2015). Idiopathic Basal Ganglia Calcification-associated *PDGFRB* Mutations Impair the Receptor Signalling. J. Cell. Mol. Med..

[167] Mathorne S.W., Sørensen K., Fagerberg C., Bode M., Hertz J.M. (2019). A Novel PDGFRB Sequence Variant in a Family with a Mild Form of Primary Familial Brain Calcification: A Case Report and a Review of the Literature. BMC Neurol..

[168] Lindblom P., Gerhardt H., Liebner S., Abramsson A., Enge M., Hellström M., Bäckström G., Fredriksson S., Landegren U., Nyström H.C. (2003). Endothelial PDGF-B Retention Is Required for Proper Investment of Pericytes in the Microvessel Wall. Genes. Dev..

[169] Watson A.N., Berthiaume A.-A., Faino A.V., McDowell K.P., Bhat N.R., Hartmann D.A., Shih A.Y. (2020). Mild Pericyte Deficiency Is Associated with Aberrant Brain Microvascular Flow in Aged PDGFRβ ^+/−^ Mice. J. Cereb. Blood Flow. Metab..

[170] Bhattacharya S.K. (2006). Focus on Molecules: Cochlin. Exp. Eye Res..

[171] Hosokawa S., Mizuta K., Nakanishi H., Hashimoto Y., Arai M., Mineta H., Shindo S., Ikezono T. (2010). Ultrastructural Localization of Cochlin in the Rat Cochlear Duct. Audiol. Neurootol..

[172] Ikezono T., Omori A., Ichinose S., Pawankar R., Watanabe A., Yagi T. (2001). Identification of the Protein Product of the Coch Gene (Hereditary Deafness Gene) as the Major Component of Bovine Inner Ear Protein. Biochim. Biophys. Acta.

[173] Mizuta K., Ikezono T., Iwasaki S., Arai M., Hashimoto Y., Pawankar R., Watanabe T., Shindo S., Mineta H. (2008). Ultrastructural Co-Localization of Cochlin and Type II Collagen in the Rat Semicircular Canal. Neurosci. Lett..

[174] Booth K.T., Ghaffar A., Rashid M., Hovey L.T., Hussain M., Frees K., Renkes E.M., Nishimura C.J., Shahzad M., Smith R.J. (2020). Novel Loss-of-Function Mutations in COCH Cause Autosomal Recessive Nonsyndromic Hearing Loss. Hum. Genet..

[175] Cho H.-J., Park H.-J., Trexler M., Venselaar H., Lee K.-Y., Robertson N.G., Baek J.-I., Kang B.S., Morton C.C., Vriend G. (2012). A Novel COCH Mutation Associated with Autosomal Dominant Nonsyndromic Hearing Loss Disrupts the Structural Stability of the vWFA2 Domain. J. Mol. Med..

[176] Danial-Farran N., Chervinsky E., Nadar-Ponniah P.T., Cohen Barak E., Taiber S., Khayat M., Avraham K.B., Shalev S.A. (2021). Homozygote Loss-of-Function Variants in the Human COCH Gene Underlie Hearing Loss. Eur. J. Hum. Genet..

[177] Grabski R., Szul T., Sasaki T., Timpl R., Mayne R., Hicks B., Sztul E. (2003). Mutations in COCH That Result in Non-Syndromic Autosomal Dominant Deafness (DFNA9) Affect Matrix Deposition of Cochlin. Hum. Genet..

[178] Shindo S., Ikezono T., Ishizaki M., Sekiguchi S., Mizuta K., Li L., Takumida M., Pawankar R., Yagi T. (2008). Spatiotemporal Expression of Cochlin in the Inner Ear of Rats during Postnatal Development. Neurosci. Lett..

[179] Ren Y., Landegger L.D., Stankovic K.M. (2019). Gene Therapy for Human Sensorineural Hearing Loss. Front. Cell Neurosci..

[180] Wu D.K., Kelley M.W. (2012). Molecular Mechanisms of Inner Ear Development. Cold Spring Harb. Perspect. Biol..

[181] Dabravolski S.A., Markin A.M., Andreeva E.R., Eremin I.I., Orekhov A.N., Melnichenko A.A. (2022). Molecular Mechanisms Underlying Pathological and Therapeutic Roles of Pericytes in Atherosclerosis. Int. J. Mol. Sci..

[182] Pak J.H., Kim Y., Yi J., Chung J.W. (2020). Antioxidant Therapy against Oxidative Damage of the Inner Ear: Protection and Preconditioning. Antioxidants.

[183] Jiang W., Zhou Z., Wang Y., Gao W., Li L., Si J. (2023). PGC-1α Affects Cochlear Pericytes Migration in Noise-Exposed Mice. Biochem. Biophys. Res. Commun..

[184] Ding D., Jiang H., Chen G.-D., Longo-Guess C., Muthaiah V.P.K., Tian C., Sheppard A., Salvi R., Johnson K.R. (2016). N-Acetyl-Cysteine Prevents Age-Related Hearing Loss and the Progressive Loss of Inner Hair Cells in γ-Glutamyl Transferase 1 Deficient Mice. Aging.

[185] Fetoni A.R., Ralli M., Sergi B., Parrilla C., Troiani D., Paludetti G. (2009). Protective Effects of N-Acetylcysteine on Noise-Induced Hearing Loss in Guinea Pigs. Acta Otorhinolaryngol. Ital..

[186] Lorito G., Giordano P., Prosser S., Martini A., Hatzopoulos S. (2006). Noise-Induced Hearing Loss: A Study on the Pharmacological Protection in the Sprague Dawley Rat with N-Acetyl-Cysteine. Acta Otorhinolaryngol. Ital..

[187] Lu J., Li W., Du X., Ewert D.L., West M.B., Stewart C., Floyd R.A., Kopke R.D. (2014). Antioxidants Reduce Cellular and Functional Changes Induced by Intense Noise in the Inner Ear and Cochlear Nucleus. J. Assoc. Res. Otolaryngol..

[188] Wang W., Li D., Ding X., Zhao Q., Chen J., Tian K., Qiu Y., Lu L. (2017). N-Acetylcysteine Protects Inner Ear Hair Cells and Spiral Ganglion Neurons from Manganese Exposure by Regulating ROS Levels. Toxicol. Lett..

[189] Pathak S., Stern C., Vambutas A. (2015). N-Acetylcysteine Attenuates Tumor Necrosis Factor Alpha Levels in Autoimmune Inner Ear Disease Patients. Immunol. Res..

[190] Castañeda R., Natarajan S., Jeong S.Y., Hong B.N., Kang T.H. (2019). Traditional Oriental Medicine for Sensorineural Hearing Loss: Can Ethnopharmacology Contribute to Potential Drug Discovery?. J. Ethnopharmacol..

[191] Ding D., Zhang J., Liu F., Li P., Qi W., Xing Y., Shi H., Jiang H., Sun H., Yin S. (2020). Antioxidative Stress-Induced Damage in Cochlear Explants. J. Otol..

[192] Hatano M., Uramoto N., Okabe Y., Furukawa M., Ito M. (2008). Vitamin E and Vitamin C in the Treatment of Idiopathic Sudden Sensorineural Hearing Loss. Acta Oto-Laryngol..

[193] García-Alcántara F., Murillo-Cuesta S., Pulido S., Bermúdez-Muñoz J.M., Martínez-Vega R., Milo M., Varela-Nieto I., Rivera T. (2018). The Expression of Oxidative Stress Response Genes Is Modulated by a Combination of Resveratrol and N-Acetylcysteine to Ameliorate Ototoxicity in the Rat Cochlea. Hear. Res..

[194] Yang Z., Zhang Y., Yang S., Ding Y., Qu Y. (2022). Low-Dose Resveratrol Inhibits RIPK3-Mediated Necroptosis and Delays the Onset of Age-Related Hearing Loss. Front. Pharmacol..

[195] Pisani A., Paciello F., Montuoro R., Rolesi R., Galli J., Fetoni A.R. (2023). Antioxidant Therapy as an Effective Strategy against Noise-Induced Hearing Loss: From Experimental Models to Clinic. Life.

[196] Anfuso C.D., Cosentino A., Agafonova A., Zappalà A., Giurdanella G., Trovato Salinaro A., Calabrese V., Lupo G. (2022). Pericytes of Stria Vascularis Are Targets of Cisplatin-Induced Ototoxicity: New Insights into the Molecular Mechanisms Involved in Blood-Labyrinth Barrier Breakdown. Int. J. Mol. Sci..

[197] Cupps T.R., Fauci A.S. (1982). Corticosteroid-Mediated Immunoregulation in Man. Immunol. Rev..

[198] Guilpain P., Le Jeunne C. (2012). Effets anti-inflammatoires et immunosuppresseurs des glucocorticoïdes. La Presse Médicale.

[199] Lee S.-H., Lyu A.-R., Shin S.-A., Jeong S.-H., Lee S.-A., Park M.J., Park Y.-H. (2019). Cochlear Glucocorticoid Receptor and Serum Corticosterone Expression in a Rodent Model of Noise-Induced Hearing Loss: Comparison of Timing of Dexamethasone Administration. Sci. Rep..

[200] Trune D.R., Kempton J.B., Gross N.D. (2006). Mineralocorticoid Receptor Mediates Glucocorticoid Treatment Effects in the Autoimmune Mouse Ear. Hear. Res..

[201] Rice J.B., White A.G., Scarpati L.M., Wan G., Nelson W.W. (2017). Long-Term Systemic Corticosteroid Exposure: A Systematic Literature Review. Clin. Ther..

[202] Heywood R.L., Hadavi S., Donnelly S., Patel N. (2013). Infliximab for Autoimmune Inner Ear Disease: Case Report and Literature Review. J. Laryngol. Otol..

[203] Wang X., Truong T., Billings P.B., Harris J.P., Keithley E.M. (2003). Blockage of Immune-Mediated Inner Ear Damage by Etanercept. Otol. Neurotol..

[204] Koller G.M., Schafer C., Kemp S.S., Aguera K.N., Lin P.K., Forgy J.C., Griffin C.T., Davis G.E. (2020). Proinflammatory Mediators, IL (Interleukin)-1β, TNF (Tumor Necrosis Factor) α, and Thrombin Directly Induce Capillary Tube Regression. Arterioscler. Thrombosis Vasc. Biol..

[205] Wong M., Ziring D., Korin Y., Desai S., Kim S., Lin J., Gjertson D., Braun J., Reed E., Singh R.R. (2008). TNFα Blockade in Human Diseases: Mechanisms and Future Directions. Clin. Immunol..

[206] Arpornchayanon W., Canis M., Ihler F., Settevendemie C., Strieth S. (2013). TNF-α Inhibition Using Etanercept Prevents Noise-Induced Hearing Loss by Improvement of Cochlear Blood Flow in Vivo. Int. J. Audiol..

[207] Tan W.J.T., Vlajkovic S.M. (2023). Molecular Characteristics of Cisplatin-Induced Ototoxicity and Therapeutic Interventions. Int. J. Mol. Sci..

[208] Geranmayeh M.H., Rahbarghazi R., Farhoudi M. (2019). Targeting Pericytes for Neurovascular Regeneration. Cell Commun. Signal.

[209] Saberianpour S., Heidarzadeh M., Geranmayeh M.H., Hosseinkhani H., Rahbarghazi R., Nouri M. (2018). Tissue Engineering Strategies for the Induction of Angiogenesis Using Biomaterials. J. Biol. Eng..

[210] Gökçinar-Yagci B., Uçkan-Çetinkaya D., Çelebi-Saltik B. (2015). Pericytes: Properties, Functions and Applications in Tissue Engineering. Stem Cell Rev. Rep..

[211] Shibuya M. (2011). Vascular Endothelial Growth Factor (VEGF) and Its Receptor (VEGFR) Signaling in Angiogenesis: A Crucial Target for Anti- and Pro-Angiogenic Therapies. Genes Cancer.

[212] Eilken H.M., Diéguez-Hurtado R., Schmidt I., Nakayama M., Jeong H.-W., Arf H., Adams S., Ferrara N., Adams R.H. (2017). Pericytes Regulate VEGF-Induced Endothelial Sprouting through VEGFR1. Nat. Commun..

[213] Gu J., Tong L., Lin X., Chen Y., Wu H., Wang X., Yu D. (2022). The Disruption and Hyperpermeability of Blood-Labyrinth Barrier Mediates Cisplatin-Induced Ototoxicity. Toxicol. Lett..

[214] Krock B.L., Skuli N., Simon M.C. (2011). Hypoxia-Induced Angiogenesis: Good and Evil. Genes Cancer.

[215] Zimna A., Kurpisz M. (2015). Hypoxia-Inducible Factor-1 in Physiological and Pathophysiological Angiogenesis: Applications and Therapies. BioMed Res. Int..

[216] Zhu H., Zhang S. (2018). Hypoxia Inducible Factor-1α/Vascular Endothelial Growth Factor Signaling Activation Correlates with Response to Radiotherapy and Its Inhibition Reduces Hypoxia-induced Angiogenesis in Lung Cancer. J. Cell. Biochem..

[217] De Souza L.E.B., Malta T.M., Kashima Haddad S., Covas D.T. (2016). Mesenchymal Stem Cells and Pericytes: To What Extent Are They Related?. Stem Cells Dev..

[218] Tashima T. (2024). Mesenchymal Stem Cell (MSC)-Based Drug Delivery into the Brain across the Blood–Brain Barrier. Pharmaceutics.

[219] Wong S.-P., Rowley J.E., Redpath A.N., Tilman J.D., Fellous T.G., Johnson J.R. (2015). Pericytes, Mesenchymal Stem Cells and Their Contributions to Tissue Repair. Pharmacol. Ther..

[220] Mannino G., Gennuso F., Giurdanella G., Conti F., Drago F., Salomone S., Furno D.L., Bucolo C., Giuffrida R. (2020). Pericyte-like Differentiation of Human Adipose-Derived Mesenchymal Stem Cells: An in Vitro Study. World J. Stem Cells.

[221] Da Silva Meirelles L., Caplan A.I., Nardi N.B. (2013). Pericytes as the Source of Mesenchymal Stem Cells. Resident Stem Cells and Regenerative Therapy.

[222] Cho Y.-B., Cho H.-H., Jang S., Jeong H.-S., Park J.-S. (2011). Transplantation of Neural Differentiated Human Mesenchymal Stem Cells into the Cochlea of an Auditory-Neuropathy Guinea Pig Model. J. Korean Med. Sci..

[223] Warnecke A., Harre J., Shew M., Mellott A.J., Majewski I., Durisin M., Staecker H. (2021). Successful Treatment of Noise-Induced Hearing Loss by Mesenchymal Stromal Cells: An RNAseq Analysis of Protective/Repair Pathways. Front. Cell. Neurosci..

[224] Morshedzadeh F., Ghanei M., Lotfi M., Ghasemi M., Ahmadi M., Najari-Hanjani P., Sharif S., Mozaffari-Jovin S., Peymani M., Abbaszadegan M.R. (2024). An Update on the Application of CRISPR Technology in Clinical Practice. Mol. Biotechnol..

[225] Delmaghani S., El-Amraoui A. (2020). Inner Ear Gene Therapies Take Off: Current Promises and Future Challenges. JCM.

[226] Zhang W., Kim S.M., Wang W., Cai C., Feng Y., Kong W., Lin X. (2018). Cochlear Gene Therapy for Sensorineural Hearing Loss: Current Status and Major Remaining Hurdles for Translational Success. Front. Mol. Neurosci..

[227] Reisinger E. (2020). Dual-AAV Delivery of Large Gene Sequences to the Inner Ear. Hear. Res..

[228] Wang D., Tai P.W.L., Gao G. (2019). Adeno-Associated Virus Vector as a Platform for Gene Therapy Delivery. Nat. Rev. Drug Discov..

[229] Akil O., Dyka F., Calvet C., Emptoz A., Lahlou G., Nouaille S., Boutet De Monvel J., Hardelin J.-P., Hauswirth W.W., Avan P. (2019). Dual AAV-Mediated Gene Therapy Restores Hearing in a DFNB9 Mouse Model. Proc. Natl. Acad. Sci. USA.

[230] Al-Moyed H., Cepeda A.P., Jung S., Moser T., Kügler S., Reisinger E. (2019). A dual-AAV Approach Restores Fast Exocytosis and Partially Rescues Auditory Function in Deaf Otoferlin Knock-out Mice. EMBO Mol. Med..

[231] Bankoti K., Generotti C., Hwa T., Wang L., O’Malley B.W., Li D. (2021). Advances and Challenges in Adeno-Associated Viral Inner-Ear Gene Therapy for Sensorineural Hearing Loss. Mol. Ther.—Methods Clin. Dev..

[232] Lahlou G., Calvet C., Giorgi M., Lecomte M.-J., Safieddine S. (2023). Towards the Clinical Application of Gene Therapy for Genetic Inner Ear Diseases. JCM.

[233] Aljabali A.A.A., El-Tanani M., Tambuwala M.M. (2024). Principles of CRISPR-Cas9 Technology: Advancements in Genome Editing and Emerging Trends in Drug Delivery. J. Drug Deliv. Sci. Technol..

[234] Li T., Yang Y., Qi H., Cui W., Zhang L., Fu X., He X., Liu M., Li P., Yu T. (2023). CRISPR/Cas9 Therapeutics: Progress and Prospects. Signal Transduct. Target. Ther..

[235] Botto C., Dalkara D., El-Amraoui A. (2021). Progress in Gene Editing Tools and Their Potential for Correcting Mutations Underlying Hearing and Vision Loss. Front. Genome Ed..

[236] Yin G., Wang X.-H., Sun Y. (2023). Recent Advances in CRISPR-Cas System for the Treatment of Genetic Hearing Loss. Am. J. Stem Cells.

[237] Ding N., Lee S., Lieber-Kotz M., Yang J., Gao X. (2021). Advances in Genome Editing for Genetic Hearing Loss. Adv. Drug Deliv. Rev..

[238] Zhang Z.-S., Liu Y.-Y., He S.-S., Bao D.-Q., Wang H.-C., Zhang J., Peng X.-Y., Zang J.-T., Zhu Y., Wu Y. (2023). Pericytes Protect Rats and Mice from Sepsis-Induced Injuries by Maintaining Vascular Reactivity and Barrier Function: Implication of miRNAs and Microvesicles. Mil. Med. Res..

[239] Mahmoudian-sani M.-R., Mehri-Ghahfarrokhi A., Ahmadinejad F., Hashemzadeh-Chaleshtori M., Saidijam M., Jami M.-S. (2017). MicroRNAs: Effective Elements in Ear-Related Diseases and Hearing Loss. Eur. Arch. Otorhinolaryngol..

[240] Ushakov K., Rudnicki A., Avraham K.B. (2013). MicroRNAs in Sensorineural Diseases of the Ear. Front. Mol. Neurosci..

[241] Zou J., Poe D., Bjelke B., Pyykkö I. (2009). Visualization of Inner Ear Disorders with MRI in Vivo: From Animal Models to Human Application. Acta Oto-Laryngol..

[242] Straka H., Zwergal A., Cullen K.E. (2016). Vestibular Animal Models: Contributions to Understanding Physiology and Disease. J. Neurol..

[243] Blanco-Sánchez B., Clément A., Phillips J.B., Westerfield M. (2017). Zebrafish Models of Human Eye and Inner Ear Diseases. Methods in Cell Biology.

[244] Friedman L.M., Dror A.A., Avraham K.B. (2007). Mouse Models to Study Inner Ear Development and Hereditary Hearing Loss. Int. J. Dev. Biol..

[245] Fukunaga I., Fujimoto A., Hatakeyama K., Aoki T., Nishikawa A., Noda T., Minowa O., Kurebayashi N., Ikeda K., Kamiya K. (2016). In Vitro Models of GJB2-Related Hearing Loss Recapitulate Ca^2+^ Transients via a Gap Junction Characteristic of Developing Cochlea. Stem Cell Rep..

[246] Ohlemiller K.K., Jones S.M., Johnson K.R. (2016). Application of Mouse Models to Research in Hearing and Balance. J. Assoc. Res. Otolaryngol..

[247] Keithley E.M., Canto C., Zheng Q.Y., Fischel-Ghodsian N., Johnson K.R. (2004). Age-Related Hearing Loss and the Ahl Locus in Mice. Hear. Res..

[248] Cuervo H., Pereira B., Nadeem T., Lin M., Lee F., Kitajewski J., Lin C.-S. (2017). PDGFRβ-P2A-CreERT2 Mice: A Genetic Tool to Target Pericytes in Angiogenesis. Angiogenesis.

[249] Johnson K.R., Tian C., Gagnon L.H., Jiang H., Ding D., Salvi R. (2017). Effects of Cdh23 Single Nucleotide Substitutions on Age-Related Hearing Loss in C57BL/6 and 129S1/Sv Mice and Comparisons with Congenic Strains. Sci. Rep..

[250] Liu S., Li S., Zhu H., Cheng S., Zheng Q.Y. (2012). A Mutation in the Cdh23 Gene Causes Age-Related Hearing Loss in Cdh23nmf308/Nmf308 Mice. Gene.

[251] Kane K.L., Longo-Guess C.M., Gagnon L.H., Ding D., Salvi R.J., Johnson K.R. (2012). Genetic Background Effects on Age-Related Hearing Loss Associated with Cdh23 Variants in Mice. Hear. Res..

[252] Baumann J., Tsao C.-C., Patkar S., Huang S.-F., Francia S., Magnussen S.N., Gassmann M., Vogel J., Köster-Hegmann C., Ogunshola O.O. (2022). Pericyte, but Not Astrocyte, Hypoxia Inducible Factor-1 (HIF-1) Drives Hypoxia-Induced Vascular Permeability in Vivo. Fluids Barriers CNS.

[253] He Y., Bao B., Li H. (2017). Using Zebrafish as a Model to Study the Role of Epigenetics in Hearing Loss. Expert. Opin. Drug Discov..

[254] Lu Z., DeSmidt A.A. (2013). Early Development of Hearing in Zebrafish. J. Assoc. Res. Otolaryngol..

[255] Vona B., Doll J., Hofrichter M.A.H., Haaf T., Varshney G.K. (2020). Small Fish, Big Prospects: Using Zebrafish to Unravel the Mechanisms of Hereditary Hearing Loss. Hear. Res..

[256] Abbas L., Whitfield T.T. (2010). The Zebrafish Inner Ear. Fish Physiology.

[257] Shi T., Beaulieu M.O., Saunders L.M., Fabian P., Trapnell C., Segil N., Crump J.G., Raible D.W. (2023). Single-Cell Transcriptomic Profiling of the Zebrafish Inner Ear Reveals Molecularly Distinct Hair Cell and Supporting Cell Subtypes. eLife.

[258] Chávez M.N., Aedo G., Fierro F.A., Allende M.L., Egaña J.T. (2016). Zebrafish as an Emerging Model Organism to Study Angiogenesis in Development and Regeneration. Front. Physiol..

[259] Burton E.A., Burgess H.A. (2023). A Critical Review of Zebrafish Neurological Disease Models−2. Application: Functional and Neuroanatomical Phenotyping Strategies and Chemical Screens. Oxf. Open Neurosci..

[260] Auer T.O., Duroure K., De Cian A., Concordet J.-P., Del Bene F. (2014). Highly Efficient CRISPR/Cas9-Mediated Knock-in in Zebrafish by Homology-Independent DNA Repair. Genome Res..

[261] Hwang W.Y., Fu Y., Reyon D., Maeder M.L., Tsai S.Q., Sander J.D., Peterson R.T., Yeh J.-R.J., Joung J.K. (2013). Efficient Genome Editing in Zebrafish Using a CRISPR-Cas System. Nat. Biotechnol..

[262] Lee H.-C., Lin C.-Y., Tsai H.-J. (2021). Zebrafish, an In Vivo Platform to Screen Drugs and Proteins for Biomedical Use. Pharmaceuticals.

[263] Wang L., Liu F., Fang Y., Ma J., Wang J., Qu L., Yang Q., Wu W., Jin L., Sun D. (2023). Advances in Zebrafish as a Comprehensive Model of Mental Disorders. Depress. Anxiety.

[264] Bahrami N., Childs S.J., Birbrair A. (2018). Pericyte Biology in Zebrafish. Pericyte Biology—Novel Concepts.

[265] Shih Y.-H., Portman D., Idrizi F., Grosse A., Lawson N.D. (2021). Integrated Molecular Analysis Identifies a Conserved Pericyte Gene Signature in Zebrafish. Development.

[266] Ando K., Fukuhara S., Izumi N., Nakajima H., Fukui H., Kelsh R.N., Mochizuki N. (2016). Clarification of Mural Cell Coverage of Vascular Endothelial Cells by Live Imaging of Zebrafish. Development.

[267] Van Der Valk W.H., Steinhart M.R., Zhang J., Koehler K.R. (2021). Building Inner Ears: Recent Advances and Future Challenges for in Vitro Organoid Systems. Cell Death Differ..

[268] Kesser B.W., Hashisaki G.T., Fletcher K., Eppard H., Holt J.R. (2007). An in Vitro Model System to Study Gene Therapy in the Human Inner Ear. Gene Ther..

[269] Warren E., Gerecht S. (2023). Beyond the Endothelium: The Role Of Mural Cells In Vascular Biology: In Vitro Systems to Study Endothelial/Pericyte Cell Interactions. Vasc. Biol..

[270] Giurdanella G., Montalbano G., Gennuso F., Brancati S., Lo Furno D., Augello A., Bucolo C., Drago F., Salomone S. (2019). Isolation, Cultivation, and Characterization of Primary Bovine Cochlear Pericytes: A New in Vitro Model of Stria Vascularis. J. Cell. Physiol..

[271] Tigges U., Welser-Alves J.V., Boroujerdi A., Milner R. (2012). A Novel and Simple Method for Culturing Pericytes from Mouse Brain. Microvasc. Res..

[272] Roccio M., Edge A.S.B. (2019). Inner Ear Organoids: New Tools to Understand Neurosensory Cell Development, Degeneration and Regeneration. Development.

[273] Rustenhoven J., Smyth L.C., Jansson D., Schweder P., Aalderink M., Scotter E.L., Mee E.W., Faull R.L.M., Park T.I.-H., Dragunow M. (2018). Modelling Physiological and Pathological Conditions to Study Pericyte Biology in Brain Function and Dysfunction. BMC Neurosci..

[274] Schneider G., Bubel M., Pohlemann T., Oberringer M. (2015). Response of Endothelial Cells and Pericytes to Hypoxia and Erythropoietin in a Co-Culture Assay Dedicated to Soft Tissue Repair. Mol. Cell Biochem..

[275] Bonkowski D., Katyshev V., Balabanov R.D., Borisov A., Dore-Duffy P. (2011). The CNS Microvascular Pericyte: Pericyte-Astrocyte Crosstalk in the Regulation of Tissue Survival. Fluids Barriers CNS.

[276] Canfield S.G., Stebbins M.J., Faubion M.G., Gastfriend B.D., Palecek S.P., Shusta E.V. (2019). An Isogenic Neurovascular Unit Model Comprised of Human Induced Pluripotent Stem Cell-Derived Brain Microvascular Endothelial Cells, Pericytes, Astrocytes, and Neurons. Fluids Barriers CNS.

[277] Faal T., Phan D.T.T., Davtyan H., Scarfone V.M., Varady E., Blurton-Jones M., Hughes C.C.W., Inlay M.A. (2019). Induction of Mesoderm and Neural Crest-Derived Pericytes from Human Pluripotent Stem Cells to Study Blood-Brain Barrier Interactions. Stem Cell Rep..

[278] Shafiee S., Shariatzadeh S., Zafari A., Majd A., Niknejad H. (2021). Recent Advances on Cell-Based Co-Culture Strategies for Prevascularization in Tissue Engineering. Front. Bioeng. Biotechnol..

[279] Dibble M., Di Cio’ S., Luo P., Balkwill F., Gautrot J.E. (2023). The Impact of Pericytes on the Stability of Microvascular Networks in Response to Nanoparticles. Sci. Rep..

[280] Kondo T., Hosoya K., Hori S., Tomi M., Ohtsuki S., Takanaga H., Nakashima E., Iizasa H., Asashima T., Ueda M. (2003). Establishment of Conditionally Immortalized Rat Retinal Pericyte Cell Lines (TR-rPCT) and Their Application in a Co-Culture System Using Retinal Capillary Endothelial Cell Line (TR-iBRB2). Cell Struct. Funct..

[281] Li P., Wu Y., Goodwin A.J., Halushka P.V., Wilson C.L., Schnapp L.M., Fan H. (2021). Generation of a New Immortalized Human Lung Pericyte Cell Line: A Promising Tool for Human Lung Pericyte Studies. Lab. Investig..

[282] Umehara K., Sun Y., Hiura S., Hamada K., Itoh M., Kitamura K., Oshima M., Iwama A., Saito K., Anzai N. (2018). A New Conditionally Immortalized Human Fetal Brain Pericyte Cell Line: Establishment and Functional Characterization as a Promising Tool for Human Brain Pericyte Studies. Mol. Neurobiol..

[283] Carter M., Shieh J. (2015). Cell Culture Techniques. Guide to Research Techniques in Neuroscience.

[284] Obinata M. (2007). The Immortalized Cell Lines with Differentiation Potentials: Their Establishment and Possible Application. Cancer Sci..

[285] Stacey G. (2006). Primary Cell Cultures and Immortal Cell Lines. Encyclopedia of Life Sciences.

[286] Harrell C.R., Simovic Markovic B., Fellabaum C., Arsenijevic A., Djonov V., Volarevic V. (2018). Molecular Mechanisms Underlying Therapeutic Potential of Pericytes. J. Biomed. Sci..

[287] Mahshid S.S., Higazi A.M., Ogier J.M., Dabdoub A. (2022). Extracellular Biomarkers of Inner Ear Disease and Their Potential for Point-of-Care Diagnostics. Adv. Sci..

[288] Enomoto N., Suzuki S., Hozumi H., Karayama M., Suzuki Y., Furuhashi K., Fujisawa T., Nakamura Y., Odagiri K., Ishikawa T. (2021). Diagnostic and Prognostic Significance of Serum Angiopoietin-1 and -2 Concentrations in Patients with Pulmonary Hypertension. Sci. Rep..

[289] Xiang D., Feng Y., Wang J., Zhang X., Shen J., Zou R., Yuan Y. (2019). Platelet-derived Growth factor-BB Promotes Proliferation and Migration of Retinal Microvascular Pericytes by Up-regulating the Expression of C-X-C Chemokine Receptor Types 4. Exp. Ther. Med..

[290] Zou J., Zhao Z., Song X., Zhang G., Li H., Zhang Q., Pyykkö I. (2022). Elevated G-CSF, IL8, and HGF in Patients with Definite Meniere’s Disease May Indicate the Role of NET Formation in Triggering Autoimmunity and Autoinflammation. Sci. Rep..

[291] Gomaa N.A., Jimoh Z., Campbell S., Zenke J.K., Szczepek A.J. (2020). Biomarkers for Inner Ear Disorders: Scoping Review on the Role of Biomarkers in Hearing and Balance Disorders. Diagnostics.

[292] Yang C.-H., Yang M.-Y., Hwang C.-F., Lien K.-H. (2023). Functional and Molecular Markers for Hearing Loss and Vertigo Attacks in Meniere’s Disease. Int. J. Mol. Sci..

[293] Sacks D., Parham K. (2015). Preliminary Report on the Investigation of the Association Between BPPV and Osteoporosis Using Biomarkers. Otol. Neurotol..

[294] Mulry E., Parham K. (2020). Inner Ear Proteins as Potential Biomarkers. Otol. Neurotol..

[295] Parham K., Sacks D., Bixby C., Fall P. (2014). Inner Ear Protein as a Biomarker in Circulation?. Otolaryngol.—Head. Neck Surg..

[296] Parham K. (2015). Prestin as a Biochemical Marker for Early Detection of Acquired Sensorineural Hearing Loss. Med. Hypotheses.

[297] Sun C., Xuan X., Zhou Z., Yuan Y., Xue F. (2020). A Preliminary Report on the Investigation of Prestin as a Biomarker for Idiopathic Sudden Sensorineural Hearing Loss. Ear Nose Throat J..

[298] Beck K., Gambee J.E., Bohan C.A., Ächinger H.P.B. (1996). The C-Terminal Domain of Cartilage Matrix Protein Assembles into a Triple-Stranded α-Helical Coiled-Coil Structure. J. Mol. Biol..

[299] Arnaud L., Mathian A., Haroche J., Gorochov G., Amoura Z. (2014). Pathogenesis of Relapsing Polychondritis: A 2013 Update. Autoimmun. Rev..

[300] Haase G.M., Prasad K.N. (2016). Oxidative Damage and Inflammation Biomarkers: Strategy in Hearing Disorders. Otol. Neurotol..

[301] Frejo L., Gallego-Martinez A., Requena T., Martin-Sanz E., Amor-Dorado J.C., Soto-Varela A., Santos-Perez S., Espinosa-Sanchez J.M., Batuecas-Caletrio A., Aran I. (2018). Proinflammatory Cytokines and Response to Molds in Mononuclear Cells of Patients with Meniere Disease. Sci. Rep..

[302] Flook M., Frejo L., Gallego-Martinez A., Martin-Sanz E., Rossi-Izquierdo M., Amor-Dorado J.C., Soto-Varela A., Santos-Perez S., Batuecas-Caletrio A., Espinosa-Sanchez J.M. (2019). Differential Proinflammatory Signature in Vestibular Migraine and Meniere Disease. Front. Immunol..

[303] Süslü N., Yılmaz T., Gürsel B. (2009). Utility of anti-HSP 70, TNF-α, ESR, Antinuclear Antibody, and Antiphospholipid Antibodies in the Diagnosis and Treatment of Sudden Sensorineural Hearing Loss. Laryngoscope.

[304] Aoki M., Asai M., Nishihori T., Mizuta K., Ito Y., Ando K. (2007). The Relevance of an Elevation in the Plasma Vasopressin Levels to the Pathogenesis of Meniere’s Attack. J. Neuroendocrinol..

[305] Kumagami H., Loewenheim H., Beitz E., Wild K., Schwartz H., Yamashita K., Schultz J., Paysan J., Zenner H.-P., Ruppersberg J.P. (1998). The Effect of Anti-Diuretic Hormone on the Endolymphatic Sac of the Inner Ear. Pflügers Arch. Eur. J. Physiol..

[306] Takeda T., Kakigi A., Saito H. (1995). Antidiuretic Hormone (ADH) and Endolymphatic Hydrops. Acta Oto-Laryngol..

[307] Aoki M., Ando K., Kuze B., Mizuta K., Hayashi T., Ito Y. (2005). The Association of Antidiuretic Hormone Levels with an Attack of Meniere’s Disease. Clin. Otolaryngol..

[308] Lim J.S., Lange M.E., Megerian C.A. (2003). Serum Antidiuretic Hormone Levels in Patients with Unilateral Meniere’s Disease. Laryngoscope.

[309] Hornibrook J., George P., Gourley J. (2011). Vasopressin in Definite Meniere’s Disease with Positive Electrocochleographic Findings. Acta Oto-Laryngol..

[310] Rüttiger L., Zimmermann U., Knipper M. (2017). Biomarkers for Hearing Dysfunction: Facts and Outlook. ORL.

[311] Serra-Millàs M. (2016). Are the Changes in the Peripheral Brain-Derived Neurotrophic Factor Levels Due to Platelet Activation?. World J. Psychiatry.

[312] Radka S.F., Hoist P.A., Fritsche M., Altar C.A. (1996). Presence of Brain-Derived Neurotrophic Factor in Brain and Human and Rat but Not Mouse Serum Detected by a Sensitive and Specific Immunoassay. Brain Res..

[313] Germanà A., Guerrera M.C., Laurà R., Levanti M., Aragona M., Mhalhel K., Germanà G., Montalbano G., Abbate F. (2020). Expression and Localization of BDNF/TrkB System in the Zebrafish Inner Ear. Int. J. Mol. Sci..

[314] De Kok Y. (1999). A Pro51Ser Mutation in the COCH Gene Is Associated with Late Onset Autosomal Dominant Progressive Sensorineural Hearing Loss with Vestibular Defects. Hum. Mol. Genet..

[315] Ikezono T., Matsumura T., Matsuda H., Shikaze S., Saitoh S., Shindo S., Hasegawa S., Oh S.H., Hagiwara Y., Ogawa Y. (2018). The Diagnostic Performance of a Novel ELISA for Human CTP (Cochlin-Tomoprotein) to Detect Perilymph Leakage. PLoS ONE.

[316] Calzada A.P., Lopez I.A., Beltran Parrazal L., Ishiyama A., Ishiyama G. (2012). Cochlin Expression in Vestibular Endorgans Obtained from Patients with Meniere’s Disease. Cell Tissue Res..

[317] DiBerardino F., Cesarani A., Hahn A., Alpini D. (2007). Viral Infection and Serum Antibodies to Heat Shock Protein 70 in the Acute Phase of Ménière’s Disease. Int. Tinnitus J..

[318] Ruckenstein M.J., Prasthoffer A., Bigelow D.C., Von Feldt J.M., Kolasinski S.L. (2002). Immunologic and Serologic Testing in Patients with Ménière’s Disease. Otol. Neurotol..

[319] Rauch S.D., Zurakowski D., Bloch D.B., Bloch K.J. (2000). Anti–Heat Shock Protein 70 Antibodies in Meniere’s Disease. Laryngoscope.

[320] Tebo A.E., Szankasi P., Hillman T.A., Litwin C.M., Hill H.R. (2006). Antibody Reactivity to Heat Shock Protein 70 and Inner Ear-Specific Proteins in Patients with Idiopathic Sensorineural Hearing Loss. Clin. Exp. Immunol..

[321] Kim Y.-R., Baek J.-I., Kim S.H., Kim M.-A., Lee B., Ryu N., Kim K.-H., Choi D.-G., Kim H.-M., Murphy M.P. (2019). Therapeutic Potential of the Mitochondria-Targeted Antioxidant MitoQ in Mitochondrial-ROS Induced Sensorineural Hearing Loss Caused by Idh2 Deficiency. Redox Biol..

[322] Vogt W. (1995). Oxidation of Methionyl Residues in Proteins: Tools, Targets, and Reversal. Free Radic. Biol. Med..

[323] Kamogashira T., Fujimoto C., Yamasoba T. (2015). Reactive Oxygen Species, Apoptosis, and Mitochondrial Dysfunction in Hearing Loss. BioMed Res. Int..

[324] Garrett A., Heibert D., Lithgow B. (2007). Electrovestibulography: The “DC” Potential Used to Separate Meniere’s Disease and Benign Paroxysmal Positional Vertigo. Proceedings of the 2007 29th Annual International Conference of the IEEE Engineering in Medicine and Biology Society.

[325] Counter S.A., Buchanan L.H. (2002). Neuro-Ototoxicity in Andean Adults With Chronic Lead and Noise Exposure. J. Occup. Environ. Med..

[326] Dewey R.S., Hall D.A., Guest H., Prendergast G., Plack C.J., Francis S.T. (2018). The Physiological Bases of Hidden Noise-Induced Hearing Loss: Protocol for a Functional Neuroimaging Study. JMIR Res. Protoc..

[327] Bing X., Liu C., Cao X., Li C., Gao X., Zhu F., Wu X., Guo N., Hu H., Xia M. (2023). Development of the Inner Ear and Regeneration of Hair Cells after Hearing Impairment. Fundam. Res..

[328] Kwan T., White P.M., Segil N. (2009). Development and Regeneration of the Inner Ear. Ann. N. Y Acad. Sci..

[329] Nist-Lund C., Kim J., Koehler K.R. (2022). Advancements in Inner Ear Development, Regeneration, and Repair through Otic Organoids. Curr. Opin. Genet. Dev..

[330] Santaolalla F., Salvador C., Martínez A., Sánchez J.M., Del Rey A.S. (2013). Inner Ear Hair Cell Regeneration: A Look from the Past to the Future. Neural Regen. Res..

[331] Lye J., Delaney D.S., Leith F.K., Sardesai V.S., McLenachan S., Chen F.K., Atlas M.D., Wong E.Y.M. (2023). Recent Therapeutic Progress and Future Perspectives for the Treatment of Hearing Loss. Biomedicines.

[332] Mittal R., Nguyen D., Patel A.P., Debs L.H., Mittal J., Yan D., Eshraghi A.A., Van De Water T.R., Liu X.Z. (2017). Recent Advancements in the Regeneration of Auditory Hair Cells and Hearing Restoration. Front. Mol. Neurosci..

[333] Beltrami A.P., Madeddu P. (2018). Pericytes and Cardiac Stem Cells: Common Features and Peculiarities. Pharmacol. Res..

[334] Tian X., Brookes O., Battaglia G. (2017). Pericytes from Mesenchymal Stem Cells as a Model for the Blood-Brain Barrier. Sci. Rep..

[335] Kasagi H., Kuhara T., Okada H., Sueyoshi N., Kurihara H. (2013). Mesenchymal Stem Cell Transplantation to the Mouse Cochlea as a Treatment for Childhood Sensorineural Hearing Loss. Int. J. Pediatr. Otorhinolaryngol..

[336] Eshraghi A.A., Ocak E., Zhu A., Mittal J., Davies C., Shahal D., Bulut E., Sinha R., Shah V., Perdomo M.M. (2020). Biocompatibility of Bone Marrow-Derived Mesenchymal Stem Cells in the Rat Inner Ear Following Trans-Tympanic Administration. JCM.

[337] Kanzaki S., Toyoda M., Umezawa A., Ogawa K. (2020). Application of Mesenchymal Stem Cell Therapy and Inner Ear Regeneration for Hearing Loss: A Review. Int. J. Mol. Sci..

[338] Tsai S.C.-S., Lin F.C.-F., Chang K.-H., Li M.-C., Chou R.-H., Huang M.-Y., Chen Y.-C., Kao C.-Y., Cheng C.-C., Lin H.-C. (2022). The Intravenous Administration of Skin-Derived Mesenchymal Stem Cells Ameliorates Hearing Loss and Preserves Cochlear Hair Cells in Cisplatin-Injected Mice: SMSCs Ameliorate Hearing Loss and Preserve Outer Hair Cells in Mice. Hear. Res..

[339] Geevarghese A., Herman I.M. (2014). Pericyte-Endothelial Crosstalk: Implications and Opportunities for Advanced Cellular Therapies. Transl. Res..

[340] Hou Z., Wang X., Cai J., Zhang J., Hassan A., Auer M., Shi X. (2018). Platelet-Derived Growth Factor Subunit B Signaling Promotes Pericyte Migration in Response to Loud Sound in the Cochlear Stria Vascularis. J. Assoc. Res. Otolaryngol..

[341] Kawamoto K., Yagi M., Stöver T., Kanzaki S., Raphael Y. (2003). Hearing and Hair Cells Are Protected by Adenoviral Gene Therapy with TGF-Β1 and GDNF. Mol. Ther..

[342] Zhao Y., Zhang L., Wang D., Chen B., Shu Y. (2022). Approaches and Vectors for Efficient Cochlear Gene Transfer in Adult Mouse Models. Biomolecules.

[343] Fukui H., Raphael Y. (2013). Gene Therapy for the Inner Ear. Hear. Res..

[344] Liu S.S., Yang R. (2022). Inner Ear Drug Delivery for Sensorineural Hearing Loss: Current Challenges and Opportunities. Front. Neurosci..

[345] Kang E., Shin J.W. (2016). Pericyte-Targeting Drug Delivery and Tissue Engineering. Int. J. Nanomed..

[346] Delaney D.S., Liew L.J., Lye J., Atlas M.D., Wong E.Y.M. (2023). Overcoming Barriers: A Review on Innovations in Drug Delivery to the Middle and Inner Ear. Front. Pharmacol..

[347] Lin Q., Guo Q., Zhu M., Zhang J., Chen B., Wu T., Jiang W., Tang W. (2022). Application of Nanomedicine in Inner Ear Diseases. Front. Bioeng. Biotechnol..

[348] Xu X., Zheng J., He Y., Lin K., Li S., Zhang Y., Song P., Zhou Y., Chen X. (2021). Nanocarriers for Inner Ear Disease Therapy. Front. Cell. Neurosci..

[349] Park J.-E., Kim W.C., Kim S.K., Ahn Y., Ha S.M., Kim G., Choi S., Yun W.S., Kong T.H., Lee S.H. (2022). Protection of Hearing Loss in Ototoxic Mouse Model Through SPIONs and Dexamethasone-Loaded PLGA Nanoparticle Delivery by Magnetic Attraction. Int. J. Nanomed..

[350] Koh H.B., Kim H.J., Kang S.-W., Yoo T.-H. (2023). Exosome-Based Drug Delivery: Translation from Bench to Clinic. Pharmaceutics.

[351] Warnecke A., Harre J., Staecker H., Prenzler N., Strunk D., Couillard-Despres S., Romanelli P., Hollerweger J., Lassacher T., Auer D. (2020). Extracellular Vesicles from Human Multipotent Stromal Cells Protect against Hearing Loss after Noise Trauma in Vivo. Clin. Transl. Med..

[352] Ahmed T.A., El-Badri N., Turksen K. (2017). Pericytes: The Role of Multipotent Stem Cells in Vascular Maintenance and Regenerative Medicine. Cell Biology and Translational Medicine, Volume 1.

[353] Okano T., Kelley M.W. (2012). Stem Cell Therapy for the Inner Ear: Recent Advances and Future Directions. Trends Amplif..

[354] Nacher-Soler G., Garrido J.M., Rodríguez-Serrano F. (2019). Hearing Regeneration and Regenerative Medicine: Present and Future Approaches. Arch. Med. Sci..

[355] Çelebi-Saltik B., Birbrair A. (2018). Pericytes in Tissue Engineering. Pericyte Biology—Novel Concepts.

[356] Kurmann L., Okoniewski M., Ogunshola O.O., Leeners B., Imthurn B., Dubey R.K. (2021). Transcryptomic Analysis of Human Brain-Microvascular Endothelial Response to -Pericytes: Cell Orientation Defines Barrier Function. Cells.

[357] Yin G.N., Shin T.Y., Ock J., Choi M.-J., Limanjaya A., Kwon M.-H., Liu F.-Y., Hong S.-S., Kang J.-H., Gho Y.S. (2022). Pericyte-derived Extracellular Vesicles-mimetic Nanovesicles Improves Peripheral Nerve Regeneration in Mouse Models of Sciatic Nerve Transection. Int. J. Mol. Med..

[358] Langlie J., Finberg A., Bencie N.B., Mittal J., Omidian H., Omidi Y., Mittal R., Eshraghi A.A. (2022). Recent Advancements in Cell-Based Models for Auditory Disorders. Bioimpacts.

[359] Lu J., Wang M., Meng Y., An W., Wang X., Sun G., Wang H., Liu W. (2023). Current Advances in Biomaterials for Inner Ear Cell Regeneration. Front. Neurosci..

